# Depression literacy, mental health literacy, and their relationship with psychological status and quality of life in patients with type 2 diabetes mellitus

**DOI:** 10.3389/fpubh.2024.1421053

**Published:** 2024-07-11

**Authors:** Alireza Jafari, Mahdi Moshki, Fatemehzahra Naddafi, Mousa Ghelichi-Ghojogh, Vajihe Armanmehr, Kimia Kazemi, Mahbobeh Nejatian

**Affiliations:** ^1^Department of Health Education and Health Promotion, School of Health, Social Development and Health Promotion Research Center, Gonabad University of Medical Sciences, Gonabad, Iran; ^2^Student Research Committee, Gonabad University of Medical Sciences, Gonabad, Iran; ^3^Neonatal and Children's Research Center, Department of Biostatistics and Epidemiology, School of Health, Golestan University of Medical Sciences, Gorgan, Iran; ^4^Social Development and Health Promotion Research Center, Gonabad University of Medical Sciences, Gonabad, Iran; ^5^Department of Clinical Psychology, Islamic Azad University, Birjand, Iran; ^6^Social Determinants of Health Research Center, Gonabad University of Medical Sciences, Gonabad, Iran

**Keywords:** mental literacy, stress, psychological status, depression, anxiety

## Abstract

**Background:**

This study was conducted to measure depression literacy (D-Lit) and mental health literacy (MHL) and to investigate their relationship with psychological status and quality of life among Iranian patients with type 2 diabetes mellitus (T2DM).

**Methods:**

This cross-sectional study was conducted in 2021 among 400 patients with T2DM in Iran. Samples were selected using proportional stratified sampling. Data collection tools comprised a demographic questionnaire, measures of MHL and D-Lit, the diabetes quality of life (DQOL) scale, and the DASS-21. After confirming the normality of the data using the Kolmogorov-Smirnov test, parametric statistical tests (such as one-way ANOVA, independent samples t-test, and Chi-Square) were used to investigate the relationship between the variables using SPSS v_22_ software. The results of continuous quantitative data are reported in the form of means and standard deviations, and qualitative data are reported in the form of absolute and relative frequencies.

**Results:**

In this study, 10.25% of the participants (*n* = 41) had severe depression, while 36.75% (*n* = 147) experienced severe anxiety. The mean (standard deviation) of MHL was 80.92 (9.16) from 130 points. Of the participants, only 1.7% (*n* = 7) did not answer any questions correctly on the D-lit scale, and only 5.8% (*n* = 23) were able to answer 15 questions or more correctly on the D-lit. MHL had a significant negative correlation with depression (*r* = −0.236), anxiety (*r* = −0.243), and stress (*r* = −0.155) (*P* < 0.001). There was a positive and significant correlation between MHL and D-Lit (*r* = 0.186) (*P* < 0.001). D-Lit had a significant negative correlation with depression (*r* = −0.192), anxiety (*r* = −0.238), and stress (*r* = −0.156) (*P* < 0.001). There was a positive and significant correlation between the ability to recognize disorders (*r* = 0.163), knowledge of self-treatment (*r* = 0.154), and DQOL (*P* < 0.001). Depression (*r* = −0.251), anxiety (*r* = −0.257), and stress (*r* = −0.203) had a significant negative correlation with DQOL (*P* < 0.001).

**Conclusion:**

MHL and D-Lit levels were found to be inadequate in patients with T2DM. These low levels of MHL and D-Lit among patients with T2DM were associated with higher levels of anxiety, depression, and stress, as well as a lower quality of life. Therefore, designing and implementing preventive programs to improve the mental health of patients with T2DM can help prevent mental disorders and ultimately improve their quality of life.

## Background

Type 2 diabetes mellitus (T2DM) is a common chronic and serious disease that threatens life, causes complications and disabilities, increases the cost of living, and reduces life expectancy (1, 2). The prevalence of global diabetes in adults is approximately 10.5% (536.6 million) and is estimated to increase in 2045 to 12.2% (783.2 million) (1). In addition, according to the report of the IDF (International Diabetes Federation) in 2019, the areas of the Middle East and North Africa had the highest prevalence of diabetes (12.2%) (3, 4). In Iran, approximately five million adults developed diabetes in 2017, and it is estimated that by 2030, 9.2 million people will have the disease (3, 5).

Diabetes and psychiatric disorders have a bidirectional association that influences one another in multiple ways and in different patterns (6). Studies have shown that anxiety, depression, stress, and distress are key psychological factors affecting diabetes, which may directly affect the development of the disease and the effectiveness of treatment (7–11). Depression is the most common psychiatric disorder in individuals with diabetes, and it worsens glycemic control and increases the risk of developing secondary complications (12, 13). Along with depression, anxiety is also common among patients with diabetes, and many studies have reported it (14–16). During psychological stress, counter-regulatory hormones such as catecholamine, a neurotransmitter, glucocorticoids, growth hormones, and glucagon are activated, which may cause poor glycemic control and functional impairment (17). Psychological stress, as a chief causative factor for psychosomatic disorders, has important effects on the development of diabetes through different pathways via behavioral and physiological mechanisms. Stress is associated with unhealthy lifestyle behaviors, such as inadequate eating in terms of food quality and quantity, low exercise levels, smoking, and alcohol abuse (18).

Health literacy (HL), including mental health literacy (MHL), is an important health predictor (19). MHL refers to the ability to obtain and maintain mental health information, understand mental disorders, treat them, reduce the stigma associated with mental disorders, and increase efficiency (20). A type of MHL is depression literacy (D-Lit), which includes personal and general knowledge of depression and belief in its treatment (21). Studies have shown that most people have inadequate MHL (22, 23). Most people do not know about psychological disorders, and they have a negative attitude toward their treatment or the effectiveness of treatment (24). However, the mental health of individuals in the community requires increased knowledge regarding mental illnesses to facilitate early diagnosis and intervention programs for mental disorders (24).

MHL has a negative correlation with depression and anxiety (25). D-Lit is a variable that can lead to increased professional help-seeking and improved mental health (26). Results: a study showed that MHL played a mediating role between psychological distress and mental help-seeking intentions (27). A study in Portugal reported that with improved MHL, stigma was reduced (28). A previous study mentioned that D-Lit had a negative correlation with depression stigma (29). MHL and D-Lit had a positive correlation with quality of life, and improving MHL could improve quality of life (30).

T2DM has a major impact on QOL in various domains, such as social, physical, and psychological wellbeing (31, 32). In Iran, some studies have been conducted in the field of HL and QOL among patients with T2DM (33, 34). According to the findings of a previous study, the QOL of patients with T2DM was predictable based on HL and self-care behaviors, and improved HL and self-care behaviors increased the QOL of patients with T2DM (35). In another study, MHL for psychiatric disorders associated with T2DM improved compatibility, thereby improving the quality of life and lifestyle of people with T2DM (36). However, no study has been conducted that surveys the D-Lit and MHL with regard to psychological status and quality of life in patients with T2DM. Therefore, the purpose of this study was to determine the status of MHL and depression literacy and their relationship with psychological status and quality of life among Iranian patients with T2DM.

## Method

This cross-sectional survey study was conducted in 2021 among 400 patients with T2DM in Gonabad city, Iran.

### Sample size

According to a previous study (37) and based on the following formula (the test power of 80%, the confidence level of 0.95%, the accuracy/d = 0.09, and the standard deviation of QOL= 0.62), the sample size required was calculated as 372. In addition, with a 10% drop rate, the sample size increased to 413 participants.


$$\begin{array}{l} n=\frac{{\left(z_{1-\frac{\alpha }{2}}{+z}_{1-\beta }\right)}^{2}\;{\left(s\right)}^{2}\;}{{\left(d\right)}^{2}}\;n=\frac{\left(7.84\right)\;{\left(0.62\right)}^{2}\;}{{\left(0.09\right)}^{2}}=372 \end{array}$$

### Sampling method

We used proportional stratified sampling to select the required sample. In the first stage, we considered Gonabad health centers to be strata. Then, from each center, patients with T2DM who met the inclusion criteria were selected via the simple random sampling method. After selecting the samples, we provided questionnaires to the eligible participants, who completed the questionnaires in a self-reporting manner. In this study, the researcher completed a questionnaire for illiterate people using an interview method. In this study, the inclusion criteria were as follows: patients had at least 1 year of residence in Gonabad city, had T2DM, had more than a year since the diagnosis of their diabetes, and were satisfied to participate in the study. People who did not answer more than 20% of the questions were excluded during the analysis phase.

### Data collection instruments

Data were collected using a demographic questionnaire and four additional scales: the MHL scale, the D-Lit scale, the diabetes quality of life (DQOL) scale, and the DASS-21 scale (depression, anxiety, and stress).

1. ***Demographic questionnaire:*** In this section, we describe the demographic details of T2D patients (marital status, sex, age, inhabitant, education level, job status, age at the onset of diabetes, and duration of diabetes).
2. ***DASS-21:*** Lovibond designed the scale to measure depression, stress, and anxiety. This scale has 21 questions and three subscales of anxiety, depression, and stress, with seven questions (38). The questions were measured on a 4-point Likert scale (did not apply to me at all, applied to me to some degree or some of the time, applied to me to a considerable degree or a good part of the time, and applied to me very much or most of the time). A lower score in each subscale indicates better depression, stress, and anxiety. In the Iranian population, the validity and reliability of this scale were assessed by Samani and Joukar, and Cronbach's alpha coefficients of depression, stress, and anxiety were 0.85, 0.87, and 0.75, respectively (39). In addition, the r value of the depression subscale of the DASS was 0.74 compared with the Beck depression inventory, and the anxiety subscale was 0.81 compared with the Beck anxiety inventory (38).
3. ***MHL scale:*** O'Connor created and evaluated the MHL scale in 2015 (40). The validity and reliability of this questionnaire in the Iranian population were assessed by Nejatian et al. in 2021 (41). The questionnaire was confirmed with 29 questions and six subscales: the ability to recognize disorders with eight questions, knowledge of self-treatment with two questions, attitudes that promote recognition or appropriate help-seeking behavior with 10 questions, knowledge of the professional help available with three questions, knowledge of risk factors and causes with two questions, and knowledge of where to seek information with four questions. The questions are measured with 5-point Likert scale and 4-point Likert scale, with the scoring range of this questionnaire being 29–130, with a higher score indicating a better MHL status. In addition, the omega-McDonald's and Cronbach's alpha coefficients for the entire questionnaire were 0.797 and 0.789 (41). The results of the MHL test-retest in the O'Connor study showed good stability (*r* = 0.797) (40).
4. ***D-Lit scale:*** This scale was created and evaluated by Griffiths et al. (42, 43), and Cronbach's alpha calculated as 0.70 (42). The validity and reliability of the D-Lit were assessed by Tehrani et al. in the Iranian population, and the Cronbach's alpha coefficient of the D-Lit was 0.890 (44). This scale has 21 questions and five subscales of knowledge about the effectiveness of available treatment methods (four questions); knowledge of psychological symptoms (five questions); knowledge about taking medications and their side effects (four questions); knowledge about cognitive-behavioral symptoms (six questions); and knowledge of disease severity with two questions. The questions were measured using a 3-point Likert scale ranging from “I don't know,” “True,” and “False.” Each correct answer received a score of 3, the incorrect answer received a score of 1, and I do not know if it received a score of 2. The questionnaire's scoring range is 21–63, with higher scores indicating better D-Lit status (44). The results of D-Lit's test-retest indicated good stability and reliability (*r* = 071) (42).
5. ***DQOL Brief Clinical Inventory scale:*** Burroughs and Partners designed this scale for people with T2DM (45). The questionnaire contains 15 questions and evaluates the QOL of patients with type 2 diabetes. The eight questions were measured using a 5-point Likert scale (completely unhappy to completely satisfied), and seven questions were measured by a 5-point Likert scale (never to always). The questionnaire's scoring range is 15–75, and a higher score indicates an appropriate DQOL status. The validity and reliability of this tool were evaluated by Mirfeizi in Iran, and the Persian version had a CVR >0.99, a CVI >0.75, and acceptable Cronbach's alpha coefficient (a = 0.75) and test-retest reliability (intraclass correlation coefficient = 0.81) (46).

### Statistical analysis

SPSS v_22_ software was used for data analysis. Before the data analysis, the normality of the data was verified using the Kolmogorov–Smirnov test, which was found to be normal. Therefore, parametric statistical tests consisting of one-way ANOVA (relationship between a quantitative variable and a qualitative variable with three or more states), an independent *t*-test (relationship between a quantitative variable and a qualitative variable with two states), and a chi-square test (relationship between qualitative variables) were used to investigate the relationship between variables. Pearson's correlation coefficient was used to evaluate the correlation between quantitative variables. Continuous parametric data are presented as mean and standard deviation, while dichotomous data are presented as absolute and relative frequencies. All tests were analyzed at a significance level of 0.05.

## Results

In this study, the response rate was 96.85%. Table 1 presents the demographic information about the participants. The mean (standard deviation) age of the patients, the age at the onset of diabetes, and the duration of the disease were 48.18 (11.69), 40.79 (9.60), and 8.38 (6.80), respectively. Most participants were men (*n* = 225) and married (*n* = 306). Other information is provided in Table 1.

**Table 1:** Characteristics of the demographic variables.

**Variables**		**(*****n*** = **400)**	
		***n***	**%**
Sex	Male	225	56.7
	Female	172	43.3
Age group	<30	20	5
	30–50	229	57.25
	>50	151	37.75
Marital status	Married	306	80.1
	Single	76	19.9
Education level	Illiterate	13	3.3
	Elementary	32	8.2
	Middle school	22	5.7
	High school	26	6.7
	Diploma	114	29.3
	Academic degree	182	46.8
Job	Housewife	89	22.94
	Employed	100	25.8
	Retired	56	14.43
	Self-employed	108	27.83
	labor	35	9
Inhabitant	Urban	307	79.9
	Rural	77	20.1
Age at the onset of diabetes	≤ 40	202	55.5
	> 40	162	44.5
Duration of diabetes mellitus	≤ 5	163	45.3
	6-10	101	28
	> 10	96	26.7
Methods of obtaining health information	Physician/health care providers	80	20.15
	Internet	137	34.5
	Newspapers/magazines	17	4.3
	Friends and acquaintances	53	13.3
	Book	15	3.8
	Radio, television, and satellite	80	20.15
	I do not know	15	3.8
Obtain information related to mental illness	Yes	300	78.8
	No	96	24.2
Methods of obtaining information related to mental illness	Physician/healthcare providers	75	24.3
	Psychologist/psychiatrist	19	6.15
	Friends and acquaintances	35	11.3
	Book	9	2.9
	Internet	129	41.75
	Radio, television, and satellite	42	13.6

Education level had a significant relationship with anxiety, and the level of anxiety was higher among those with elementary education, whereas those with academic education had a lower level of anxiety (*P* = 0.002). There was a significant relationship between receiving mental health information and depression levels, with those who received mental health information having lower depression levels (*P* = 0.014). There was a significant relationship between methods of obtaining information about mental health and anxiety, and those who received information from their friends and acquaintances had lower anxiety levels (*P* = 0.001). There was a significant relationship between the method of obtaining information about mental health and stress, and those who received their information from physicians/health care providers had lower stress levels (*P* = 0.042).

Age had a significant relationship with depression (*P* = 0.046), stress (*P* = 0.001), and anxiety (*P* < 0.001), whereas people younger than 30 years had lower levels of depression, stress, and anxiety. Job status had a significant relationship with anxiety, and self-employed individuals had low levels of anxiety (*P* = 0.023). Job status had a significant relationship with stress, and individuals with employed jobs experienced low levels of stress (*P* = 0.021). The age at the onset of diabetes had a significant relationship with stress (*P* = 0.009) and anxiety (*P* = 0.026), and people who had diabetes at the age of 40 years or less experienced low levels of stress and anxiety. The duration of diabetes had a significant relationship with depression, stress, and anxiety, and individuals with a duration of diabetes of 5 years had low levels of depression (*P* = 0.036), stress (*P* = 0.004), and anxiety (*P* < 0.001) (Table 2).

**Table 2:** Relationship between demographic variables and psychological status (depression, anxiety, and stress).

**Variables**		**DASS-21;** ***Mean (SD)***					
		**Depression**	***P*** **-value**	**Anxiety**	***P*** **-value**	**Stress**	***P*** **-value**
Sex^*^	Male	15.29 (3.83)	0.194	13.01 (3.45)	0.704	16.35 (4.01)	0.087
	Female	15.80 (3.90)		13.23 (3.38)		17.04 (3.79)	
Age group^**^	<30	14.25 (4.20)	0.046	11.40 (3.28)	<0.001	15.50 (4.47)	0.001
	30–50	15.27 (3.82)		12.68 (3.17)		16.13 (3.62)	
	>50	16.07 (3.92)		14.13 (3.52)		17.62 (4.26)	
Marital status^*^	Married	15.66 (3.75)	0.574	13.25 (3.28)	3.13	16.76 (3.84)	0.737
	Single	15.38 (4.27)		12.81 (3.67)		16.59 (4.24)	
Education level^**^	Illiterate	14.46 (3.57)	0.193	13.76 (3.87)	0.002	16.76 (4.63)	0.062
	Elementary	15.81 (3.60)		14.93 (3.52)		18.40 (3.09)	
	Secondary	16.09 (3.51)		14.09 (3)		17.40 (4.14)	
	High school	14.23 (3.78)		12.69 (3.42)		15.92 (3.89)	
	Diploma	16 (3.94)		13.24 (3.44)		16.71 (3.94)	
	Academic	15.20 (3.87)		12.50 (3.13)		16.18 (3.97)	
Job^**^	Housewife	15.95 (4.10)	0.253	13.19 (3.38)	0.023	17.01 (3.74)	0.021
	Employed	15.78 (4.21)		12.94 (3.63)		15.92 (4.06)	
	Retired	15.91 (3.65)		14.53 (3.12)		17.87 (4.35)	
	Self-employed	14.87 (3.76)		12.81 (3.26)		16.24 (3.83)	
	labor	15.17 (3.16)		12.85 (2.65)		17 (3.26)	
Inhabitant ^*^	Rural	15.85 (3.67)	0.491	13.09 (2.96)	0.876	16.75 (3.55)	0.842
	Urban	15.51 (3.99)		13.15 (3.55)		16.75 (4.11)	
Age at the onset of diabetes ^*^	≤ 40	15.58 (3.93)	0.960	12.95 (3.17)	0.026	13.74 (3.61)	0.009
	>40	15.60 (3.98)		13.74 (3.61)		16.21 (3.73)	
Duration of diabetes ^**^	≤ 5	15.15 (4.18)	0.036	12.79 (3.38)	<0.001	16.28 (3.84)	0.004
	6–10	15.56 (3.89)		12.89 (3.22)		16.32 (4.06)	
	>10	16.46 (3.50)		14.69 (3.23)		17.86 (3.82)	
Obtain information related to mental illness^*^	Yes	15.24 (3.83)	0.014	13.01 (3.43)	0.160	16.60 (4.04)	0.830
	No	16.35 (3.89)		13.57 (3.32)		16.76 (3.79)	
Methods of obtaining health information^**^	Physician/healthcare providers	15.38 (3.45)	0.131	13.63 (3.27)	<0.001	16.43 (3.97)	0.025
	Internet	14.97 (3.87)		12.15 (3.31)		16 (3.96)	
	Newspapers/magazines	17.41 (2.37)		15 (2.87)		18.29 (2.86)	
	Friends and acquaintances	15.92 (4.14)		13.26 (3.17)		16.69 (3.90)	
	Book	15.80 (4.12)		12.93 (3.80)		18.40 (4.48)	
	Radio, television, and satellite	16.10 (4.07)		14.12 (3.30)		17.56 (3.67)	
	I do not know	14.80 (4.63)		12.33 (4.08)		16 (5.05)	
Methods of obtaining information related to mental illness^**^	Physician/healthcare providers	14.96 (3.16)	0.195	13.78 (3.37)	0.001	16.48 (4.03)	0.042
	Psychologist/psychiatrist	16.10 (3.01)		13.84 (2.96)		17.05 (3.20)	
	Friends and acquaintances	15.42 (4.62)		12.11 (3.19)		16.57 (4.90)	
	Book	16.66 (4.41)		13.11 (3.62)		18 (3.27)	
	Internet	15.35 (4.04)		12.55 (3.51)		16.58 (3.96)	
	Radio, television, and satellite	16.76 (3.86)		14.76 (3.08)		18.73 (3.26)	

^*^Independent sample t-test, ^**^one-way ANOVA.

The mean (standard deviation) of MHL was 80.92 (9.16). Marital status had a significant relationship with MHL, and single individuals had a higher MHL (*P* = 0.009). The level of education had a significant relationship with knowledge of where to seek information, and those with higher education levels had a higher HL on this subscale (*P* < 0.001). The economic situation had a significant relationship with MHL, and patients with better economic conditions had higher MHL (*P* < 0.001). The economic situation had a significant relationship with knowledge of where to seek information, and those with better economic conditions had higher HL scores in this subscale (*P* < 0.001) (Table 3).

**Table 3:** Relationship between demographic variables and mental health literacy.

**Variables**		**Mental health literacy (MHL)**													
		***Mean (SD)***													
		**Ability to recognizedisorders**	***P*** **-value**	**Knowledge of riskfactors and causes**	***P*** **-value**	**Knowledge ofself-treatment**	***P*** **-value**	**Knowledge of the professional help available**	***P*** **-value**	**Knowledge of where to seek information**	***P*** **-value**	**Attitudes that promote recognition or appropriate help-seeking behavior**	***P*** **-value**	**Total MHL**	***P*** **-value**
Sex^*^	Male	22.99 (4.04)	0.727	5.55 (1.53)	0.167	5.68 (1.21)	0.943	9.28 (1.47)	0.143	12.71 (3.60)	0.057	24.61 (5.88)	0.868	80.85 (6.14)	0.820
	Female	22.85 (3.77)		5.33 (1.69)		5.69 (1.23)		9.06 (1.43)		13.40 (3.46)		24.71 (6.14)		81.06 (8.75)	
Age group^**^	<30	24.50 (3.20)	0.002	5.55 (1.73)	0.878	6.15 (1.34)	0.012	9.65 (1.03)	0.354	14.35 (4.22)	<0.001	23.60 (6.65)	0.541	83.80 (10.16)	<0.001
	30-50	23.31 (3.56)		5.41 (1.66)		5.78 (1.28)		9.17 (1.45)		14.02 (3.10)		24.57 (5.79)	82.29 (8.84)		
	>50	22.09 (4.37)		5.48 (1.53)		5.47 (1.09)		9.16 (1.50)		11.23 (3.41)		25.03 (6.25)	78.48 (9.04)		
Marital status^*^	Married	22.63 (3.90)	0.040	5.44 (1.58)	0.697	5.59 (1.16)	0.053	9.10 (1.45)	0.278	12.76 (3.49)	0.077	24.39 (5.71)	0.490	79.93 (8.74)	0.009
	Single	23.64 (3.68)		5.52 (1.73)		5.89 (1.33)		9.30 (1.41)		13.56 (3.66)		24.95 (6.51)		82.90 (9)	
Education level^**^	Illiterate	22 (5.79)	0.176	5.53 (2.25)	0.871	5.92 (1.32)	0.032	9.38 (2.53)	0.106	10.30 (2.46)	<0.001	25.84 (6.25)	0.761	79 (7.04)	0.429
	Elementary	23.77 (5.31)		5.25 (1.74)		5.83 (1.13)		9.69 (1.42)		9.46 (3.34)		24.29 (6.86)		78.32 (11.88)	
	Secondary	22.04 (4.02)		5.50 (2.04)		5.22 (1.06)		9.36 (1.59)		12.45 (3.69)		26.45 (6.78)		81.04 (9.26)	
	High school	24.34 (3.88)		5.50 (1.67)		6.07 (1.46)		9.23 (1.14)		13.11 (4.02)		24.33 (5.95)		82.60 (10.47)	
	Diploma	22.60 (3.42)		5.61 (1.50)		5.47 (1.07)		8.91 (1.40)		13.98 (3.03)		24.56 (5.70)		81.16 (8.76)	
	Academic	23.05 (3.77)		5.40 (1.56)		5.82 (1.27)		9.27 (1.39)		13.29 (3.46)		24.73 (5.95)		81.60 (8.70)	
Job^**^	Housewife	22.94 (3.73)	0.738	5.50 (3.88)	0.887	5.69 (1.19)	0.518	8.94 (1.44)	0.252	13.50 (3.35)	<0.001	23.77 (6.05)	0.214	80.36 (8.87)	0.010
	Employed	22.80 (3.50)		5.52 (1.65)		5.72 (1.27)		9.08 (1.53)		13.93 (3.22)		24.36 (6.42)		81.43 (9.33)	
	Retired	22.79 (4.85)		5.49 (1.79)		5.50 (1.17)		9.42 (1.52)		10.46 (3.49)		24.19 (6.09)		77.88 (8.81)	
	Self-employed	23.15 (3.76)		5.31 (1.59)		5.76 (1.28)		9.31 (1.41)		13.44 (3.40)		25.65 (5.34)		82.65 (8.93)	
	labor	22.11 (4.07)		5.40 (1.35)		5.42 (1.03)		9.25 (1.37)		11.28 (3.18)		25.17 (5.88)		78.65 (8.813)	
Inhabitant ^*^	Rural	22.93 (3.74)	0.872	5.46 (1.61)	0.952	5.68 (1.07)	0.848	9.06 (1.47)	0.548	12.50 (3.43)	0.224	0.509	0.515	80.57 (7.55)	0.949
	Urban	22.84 (3.84)		5.47 (1.59)		5.65 (1.23)		9.17 (1.43)		13.06 (3.59)		24.42 (5.94)		80.64 (9.37)	
Age at the onset of diabetes ^*^	≤ 40	23.11 (3.61)	0.086	5.44 (1.58)	0.593	5.64 (1.26)	0.607	9.09 (1.45)	0.569	13.71 (3.17)	<0.001	24.43 (5.86)	0.372	81.45 (9.16)	0.040
	>40	22.41 (4.14)		5.53 (1.59)		5.57 (1.14)		9.18 (1.47)		11.77 (3.69)		24.99 (6.09)		79.48 (9.01)	
Duration of diabetes ^**^	≤ 5	23.14 (3.68)	0.235	5.37 (1.48)	0.558	5.72 (1.23)	0.360	9.10 (1.40)	0.743	13.67 (3.37)	<0.001	24.86 (5.42)	0.864	81.88 (8.87)	0.026
	6–10	22.32 (3.640		5.57 (1.70)		5.51 (1.25)		9.08 (1.43)		13.15 (3.05)		24.51 (6.04)		80.16 (8.87)	
	>10	22.68 (4.35)		5.53 (1.62)		5.56 (1.13)		9.23 (1.61)		11.21 (3.81)		24.53 (6.65)		78.77 (9.61)	
Economic status^**^	Good	23.13 (3.50)	0.526	5.31 (1.82)	0.372	5.83 (1.28)	0.278	9.20 (1.61)	0.609	14.26 (3.68)	<0.001	26.40 (5.95)	0.007	84.15 (9.42)	<0.001
	Medium	22.6 (4.07)		5.53 (1.49)		5.59 (1.20)		9.10 (1.32)		12.88 (3.35)		24.11 (5.70)		79.87 (8.89)	
	Weak	22.49 (4.09)		5.28 (1.47)		5.69 (1.06)		9.30 (1.54)		11.24 (3.04)		24.22 (5.92)		78.25 (8.19)	
Obtain information related to mental illness^**^	Yes	23.36 (3.84)	0.001	5.43 (1.71)	0.713	5.79 (1.31)	0.001	9.29 (1.47)	0.015	13.13 (3.60)	0.177	24.81 (6.18)	0.419	81.83 (9.27)	0.001
	No	21.66 (3.89)		5.49 (1.28)		5.38 (0.87)		8.88 (1.37)		12.57 (3.42)		24.23 (5.51)		78.25 (8.36)	
Methods of obtaining health information^**^	Physician/ health care providers	23.91 (3.65)	<0.001	5.56 (1.83)	0.399	5.54 (1.19)	<0.001	9.18 (1.78)	0.949	13.07 (3.45)	<0.001	25.30 (6.60)	0.001	82.58 (8.62)	<0.001
	Internet	23.68 (3.64)	5.59 (1.67)		6.09 (1.28)	9.27 (13.1)		14.01 (3.35)		24.47 (6.26)		83.14 (9.63)			
	Newspapers/ magazines	20.05 (2.68)	5.76 (1.52)		5.64 (1.05)	9.23 (1.82)		11.05 (2.65)		27.45 (5.68)		79.22 (6.80)			
	Friends and acquaintances	23.18 (3.50)	5.45 (1.59)		5.32 (1.05)	9 (1.41)		12.69 (3.52)		24.09 (5.04)		79.74 (7.36)			
	Book	19.66 (3.67)	5 (1.51)		5.73 (0.59)	9.13 (1.50)		8.86 (2.77)		29.98 (4.42)		78.38 (4.93)			
	Radio, television, and satellite	22.33 (4.45)	5.17 (1.43)		5.44 (1.27)	9.26 (1.29)		13.13 (3.48)		23.26 (5.05)		78.62 (9.86)			
I do not know	20.12 (2.84)	5.13 (0.91)	5.40 (0.98)	9.06 (1.43)	10.13 (2.97)	24.13 (6.83)	73.99 (8.60)
Methods of obtaining information related to mental illness^**^	Physician/ health care providers	24.34 (4.17)	0.009	5.97 (1.78)	0.005	5.94 (1.44)	0.069	9.50 (1.71)	0.121	12.67 (3.96)	0.334	25.22 (6.41)	0.486	83.67 (9.17)	0.014
	Psychologist/ Psychiatrist	24.05 (3.30)		5.57 (1.67)		5.42 (0.83)		8.78 (1.47)		13.57 (3.54)		26.48 (5.46)		83.90 (10.38)	
	Friends and acquaintances	22.79 (3.07)		5.62 (1.86)		5.54 (1.19)		8.97 (1.40)		12.60 (3.27)		24.42 (6.09)		79.96 (6.80)	
	Book	21.93 (2.35)		4.44 (1.81)		5.33 (1)		10 (0.86)		13 (3.93)		27.97 (6.37)		82.69 (7.63)	
	Internet	23.09 (3.67)		5.15 (1.59)		5.94 (1.32)		9.24 (1.38)		13.43 (3.47)		24.79 (6.09)		81.66 (9.16)	
	Radio, television, and satellite	21.71 (4.49)		5.09 (1.33)		5.40 (1.25)		9.02 (1.38)		12.14 (3.58)		24.21 (6.06)		77.59 (9.84)	

^*^Independent sample t-test, ^**^ One-way ANOVA.

There was a significant relationship between receiving mental health information and the level of MHL, with those who received mental health information having higher levels of MHL (*P* = 0.001). There was a significant relationship between receiving mental health information and the ability to recognize disorders (*P* = 0.001), knowledge of self-treatment (*P* = 0.001), and knowledge of the professional help available (*P* = 0.015), and those who received mental health information in these subscales had higher levels of MHL. Similarly, patients who received health information online had a higher MHL (*P* < 0.001). Patients who received mental health information from a psychologist/psychiatrist had higher levels of MHL (*P* = 0.014) (Table 3).

Age was significantly associated with MHL, and patients younger than 30 years had higher levels of MHL (*P* < 0.001). Job status had a significant relationship with MHL, and self-employed individuals had higher levels of MHL (*P* = 0.010). Age at the onset of diabetes had a significant relationship with MHL, and patients who had diabetes at the age of 40 years or less had higher levels of MHL (*P* = 0.040). The duration of diabetes was significantly associated with MHL, and patients with a duration of diabetes of ≤ 5 years had higher levels of MHL (*P* = 0.026) (Table 3).

Age had a significant relationship with D-Lit, and people younger than 30 years had a higher D-Lit (*P* = 0.002). Job status had a significant relationship with D-Lit, and self-employed individuals had higher levels of D-Lit (*P* = 0.029). Age at the onset of diabetes had a significant relationship with D-Lit, and individuals who had a disease at the age of ≤ 40 years had higher levels of D-Lit (*P* = 0.044). There was a significant relationship between receiving mental health information and D-Lit levels, with those who received mental health information having higher levels of D-Lit (*P* = 0.001). Patients who received health information from the book had higher levels of D-Lit (*P* < 0.001). Patients who received mental health information from the book had higher D-Lit scores (*P* = 0.002) (Table 4).

**Table 4:** Frequency distribution of demographic factors and depression literacy (D-lit) status.

**Variables**		**D-lit status*****; n*** **(%)**					**D-Lit**	
		**Incorrect response/ don't know**	**The correct response to 1–7 questions**	**The correct response to 8–14 questions**	**The correct response to 15 questions or more**	**P-value** ^c^	**Mean (SD)**	***P*** **-value**
Sex	Male	3 (1.35)	64 (28.4)	146 (64.9)	12 (5.35)	0.599	44.63 (4.26)	0.837^a^
	Female	4 (2.3)	56 (32.6)	101 (58.7)	11 (6.4)		44.72 (4.32)	
Age group	<30	0	5 (25)	14 (70)	1 (5)	0.235	46.40 (4.84)	0.002^b^
	30-50	2 (0.9)	62 (27.1)	149 (65)	16 (7)		45.13 (4.08)	
	>50	5 (3.3)	53 (35.1)	87 (57.6)	6 (4)		43.80 (4.36)	
Job	Housewife	2 (2.25)	36 (40.45)	47 (52.8)	4 (4.5)	0.289	44.17 (4.23)	0.029^b^
	Employed	2 (2)	26 (26)	65 (65)	7 (7)		45.16 (4.22)	
	Retired	0	20 (35.7)	33 (58.9)	3 (5.4)		43.55 (4.95)	
	Self-employed	3 (2.8)	24 (22.2)	73 (67.6)	8 (7.4)		45.48 (4.19)	
	labor	0	11 (31.4)	24 (68.6)	0		44.05 (3.62)	
Marital status	Married	5 (1.6)	97 (31.7)	188 (61.45)	16 (5.25)	0.261	44.30 (4.30)	0.661^a^
	Single	2 (2.6)	16 (21.1)	55 (72.4)	3 (3.9)		44.89 (3.90)	
Education level	Illiteracy	0	4 (30.8)	8 (61.5)	1 (7.7)	0.108	43 (4.12)	0.001^b^
	Elementary	1 (3.1)	16 (50)	14 (43.8)	1 (3.1)		42.34 (4.17)	
	middle school	0	8 (36.4)	14 (63.6)	0		44 (5.15)	
	High school	1 (3.85)	9 (34.6)	15 (57.7)	1 (3.85)		44.92 (3.77)	
	Diploma	1 (0.8)	41 (36)	66 (57.9)	6 (5.3)		44.34 (4.25)	
	Academic	4 (2.2)	36 (19.8)	128 (70.3)	14 (7.7)		45.56 (4.07)	
Age at the onset of diabetes	≤ 40	3 (1.5)	60 (29.7)	124 (61.4)	15 (7.4)	0.437	44.95 (4.29)	0.044 ^a^
	>40	4 (2.5)	51 (31.5)	101 (62.3)	6 (3.7)		44.05 (4.08)	
Duration of diabetes mellitus	≤ 5	4 (2.4)	41 (25.2)	110 (67.5)	8 (4.9)	0.301	44.95 (3.94)	0.064 ^b^
	6-10	1 (1)	32 (31.7)	60 (59.4)	8 (7.9)		44.57 (4.13)	
	>10	2 (2.1)	37 (38.5)	52 (54.2)	5 (5.2)		43.68 (4.66)	
Economic status	Good	2 (2.2)	23 (25.6)	57 (63.3)	8 (8.9)	0.840	45.22 (4.09)	0.080^b^
	Medium	4 (2)	63 (30.7)	128 (62.4)	10 (4.9)		44.60 (4.16)	
	Weak	1 (1.5)	22 (33.3)	39 (59.1)	4 (6.1)		44.34 (4.79)	
Obtain information related to mental illness	Yes	5 (1.6)	86 (28.7)	188 (62.7)	21 (7)	0.304	45.10 (4.35)	0.001^a^
	No	2 (2.1)	32 (33.3)	60 (62.5)	2 (2.1)		43.50 (3.83)	
Inhabitant	Rural	1 (1.3)	23 (29.9)	51 (66.2)	2 (2.6)	0.612	43.94 (3.86)	0.072^a^
	Urban	6 (1.9)	93 (30.3)	189 (61.6)	19 (6.2)		44.92 (4.31)	
Methods of obtaining health information	Physician/ health care providers	1 (1.2)	32 (40)	44 (55)	3 (3.8)	0.002	43.90 (4.26)	<0.001^b^
	Internet	1 (0.7)	29 (21.2)	90 (65.7)	17 (12.4)		45.67 (4.58)	
	Newspapers/ magazines	0	4 (23.5)	13 (76.5)	0		44.47 (3.02)	
	Friends and acquaintances	2 (3.8)	20 (37.7)	31 (58.5)	0		43.45 (2.61)	
	Book	0	0	15 (100)	0		46.46 (3.71)	
	Radio, television, and satellite	2 (2.5)	28 (35)	47 (58.75)	3 (3.75)		44.96 (4.52)	
	I do not know	1 (6.7)	6 (40)	8 (53.3)	0		41.53 (3.92)	
Methods of obtaining information related to mental illness	Physician/ health care providers	0	25 (33.3)	45 (60)	5 (6.7)	0.002	44.09 (4.41)	0.002^b^
	Psychologist/ Psychiatrist	1 (5.25)	5 (26.3)	12 (63.2)	1 (5.25)		44.31 (4.11)	
	Friends and acquaintances	0	11 (31.4)	24 (68.6)	0		44.51 (3.28)	
	Book	0	1 (11.1)	8 (88.9)	0		46.66 (4.06)	
	Internet	4. (3.1)	22 (17)	89 (69)	14 (10.9)		46.13 (4.38)	
	Radio, television, and satellite	0	23 (54.7)	18 (42.9)	1 (2.4)		43.71 (3.90)	
All participants		7 (1.7)	120 (30)	250 (62.5)	23 (5.8)	-		

^a^Independent sample t-test, ^b^one-way ANOVA, ^c^ Chi-square.

In response to the D-Lit questions, only 1.7% (*n* = 7) of the participants failed to answer any questions correctly, and 5.8% (*n* = 23) were able to answer 15 questions or more correctly (Table 4). The participants' responses to the questions and subscales of the D-Lit are listed in Table 5. The mean (SD) D-Lit of the participants was 44.69 (4.28). The mean (SD) of the subscales of the D-Lit are presented in Table 5.

**Table 5:** Participants' responses to the D-lit questions.

**Subscale**	**Items**	***n*** **(%)**			**Mean (SD)**	
		**Correct response**	**Incorrect response**	**Don't know**		
F1: Knowledge of the psychological symptoms	1. People with depression may feel guilty when they are innocent. (True)	264 (66)	67 (16.8)	69 (17.2)	11.46 (2.75)	44.69 (4.28)
	2. Loss of confidence and low self-esteem may be signs of depression. (True)	236 (259.15)	101 (25.31)	62 (15.54)	
	3. Too little or too much sleep can be a symptom of depression. (True)	236 (59.1)	83 (20.8)	80 (20.1)	
	4. Eating too much or losing interest in food may be a symptom of depression. (True)	175 (43.8)	125 (31.2)	100 (25)	
	5. People may move more slowly or become agitated due to their depression. (True)	172 (43)	119 (29.75)	109 (27.25)	
F2: Knowledge of the effectiveness of available treatment methods	6. Clinical psychologists can prescribe antidepressant medications. (False)	159 (39.75)	128 (32)	113 (28.25)	8.05 (1.57)
	7. Many treatments for depression are more effective than antidepressant medications. (False)	154 (38.5)	134 (33.5)	112 (28)	
	8. The effects of counseling are similar to those of cognitive-behavioral therapies for depression. (False)	124 (31.1)	158 (39.6)	117 (29.3)	
	9. The effect of cognitive-behavioral therapies is the same as that of antidepressant medications for mild to moderate depression. (True)	143 (36)	129 (32.5)	125 (31.5)	
F3: Knowledge of cognitive-behavioral symptoms	10. People with depression often speak sporadically and irrelevantly. (False)	100 (25.1)	177 (44.5)	121 (30.4)	11.96 (2.75)
	11. Reckless and foolhardy behavior is a common symptom of depression. (False)	121 (30.4)	148 (37.2)	129 (32.4)	
	12. Not walking on cracked and broken sidewalks may be a symptom of depression. (False)	126 (31.7)	141 (35.4)	131 (32.9)	
	13. People with depression often hear sounds that are not normally heard. (False)	135 (34.1)	139 (35.1)	122 (30.8)	
	14. Depression does not affect your memory or concentration. (False)	214 (53.6)	96 (24.1)	89 (22.3)	
	15. Having several distinct personalities can be a symptom of depression. (False)	150 (37.5)	136 (34)	114 (28.5)	
F4: Knowledge of taking medications and their side effects	16. Of all the alternative and lifestyle therapies for depression, vitamins are the most beneficial. (False).	158 (39.5)	121 (30.25)	121 (30.25)	8.79 (2.01)
	17. People with depression should stop taking antidepressant medications as soon as they feel better. (False)	212 (53.1)	82 (20.6)	105 (26.3)	
	18. Antidepressant medications are addictive. (False)	185 (46.5)	118 (29.6)	95 (23.9)	
	19. Antidepressant medications are usually rapid-acting. (False)	183 (45.9)	93 (23.3)	123 (30.8)	
F5: Knowledge of disease severity	20. Most people with depression need to be hospitalized. (False)	202 (50.5)	75 (18.75)	123 (30.75)	4.42 (0.97)
	21. Many celebrities have suffered from depression. (True)	146 (36.5)	105 (26.25)	149 (37.25)	

Regarding the subscales of the D-Lit questionnaire, the responses revealed varied levels of knowledge among the participants. Specifically, only 21.5% (*n* = 86) could correctly answer all items related to knowledge of psychological symptoms. Only 2% (*n* = 8) correctly answered all questions about the effectiveness of available treatment methods. A slightly higher percentage, 4.5% (*n* = 18), correctly answered all questions pertaining to cognitive-behavioral symptoms. The participants' knowledge about taking medications and their side effects was accurately known by 13% (*n* = 52). Furthermore, 19.3% (*n* = 77) were able to correctly answer all questions regarding knowledge about disease severity.

There was a significant relationship between the sources of mental health information and the DQOL. Specifically, those who received information from their friends and acquaintances reported a higher DQOL (*P* = 0.042) (Table 6). Additionally, the results of Tukey's *post hoc* tests exploring the relationships between demographic factors and variables such as depression, anxiety, stress, MHL, D-Lit, and DQOL are presented in Supplementary Files S1–S6.

**Table 6:** Relationship between demographic variables and diabetes quality of life (DQOL).

**Variables**		**DQOL**	
		***Mean (SD)***	***P*-value**
Sex^*^	Male	55.27 (7.44)	0.152
	Female	54.20 (7.11)	
Age group^**^	<30	55.77 (11.66)	0.821
	30–50	54.85 (7.03)	
	>50	54.68 (7.12)	
Marital status^*^	Married	54.65 (7.19)	0.466
	Single	55.35 (7.59)	
Education level^**^	Illiterate	57 (7.10)	0.111
	Elementary	54.81 (6.65)	
	Secondary	54.75 (7.27)	
	High school	55.26 (7.32)	
	Diploma	53.39 (7.65)	
	Academic	55.81 (7.03)	
Job^**^	Housewife	53.91 (7.10)	0.143
	Employed	54.80 (7.83)	
	Retired	56.51 (6.51)	
	Self-employed	54.41 (7.41)	
	labor	56.69 (7.25)	
Inhabitant ^*^	Rural	55.36 (6.59)	0.532
	Urban	54.77 (7.59)	
Age the onset of diabetes ^*^	≤ 40	54.61 (7.48)	0.928
	>40	54.68 (6.70)	
Duration of diabetes ^**^	≤ 5	55.27 (6.70)	0.343
	6–10	54.01 (7.73)	
	>10	54.44 (7.10)	
Obtain information related to mental illness^*^	Yes	54.84 (7.38)	0.946
	No	54.79 (7.28)	
Methods of obtaining health information^**^	Physician/health care providers	56.33 (6.91)	0.050
	Internet	55.51 (7.02)	
	Newspapers/ magazines	54.47 (7.07)	
	Friends and acquaintances	54.22 (6.89)	
	Book	55.36 (7.53)	
	Radio, television, and satellite	52.59 (7.93)	
	I do not know	55.27 (8.88)	
Methods of obtaining information related to mental illness^**^	Physician/Health care providers	55.40 (7.06)	0.042
	Psychologist/ Psychiatrist	53.75 (6.59)	
	Friends and acquaintances	56.76 (6.84)	
	Book	55.83 (6.75)	
	Internet	54.52 (6.89)	
	Radio, television, and satellite	51.68 (8.61)	

^*^Independent sample t-test, ^**^one-way ANOVA.

As can be observed from Table 7, only 10.3% (*n* = 41) had severe depression, 36.8% (*n* = 147) had severe anxiety and extremely severe anxiety, and 31% (*n* = 124) had moderate stress. Depression (*P* = 0.010), anxiety (*P* < 0.001), and stress (*P* = 0.008) were significantly associated with D-Lit, whereas patients with severe depression, extremely severe anxiety, and moderate stress had lower levels of D-Lit. Depression (*P* < 0.001), anxiety (*P* < 0.001), and stress (*P* = 0.001) were significantly associated with MHL, and patients with severe depression, extremely severe anxiety, and moderate stress had lower levels of MHL. In addition, depression (*P* < 0.001), anxiety (*P* < 0.001), and stress (*P* = 0.002) were significantly associated with DQOL, and patients with severe depression, extremely severe anxiety, and moderate stress had lower levels of DQOL (Table 7).

**Table 7:** Relationship between depression, anxiety, and stress with D-Lit, MHL, and DQOL.

**Variables**		***n* %**	***Mean (SD)***					
			**D-Lit**	***P*** **-value**	**MHL**	***P*** **-value**	**DQOL**	***P*** **-value**
**Depression**	Normal	31 (7.75)	46 (4.6)	0.010	81.55 (11.72)	<0.001	58.93 (7.480)	<0.001
	Mild	78 (19.5)	45.79 (4.73)		84.34 (9.18)		55.97 (7.14)	
	Moderate	250 (62.5)	44.31 (4.02)		80.70 (8.52)		54.66 (7.09)	
	Severe	41 (10.25)	43.92 (4.23)		75.34 (8.10)		50.64 (7.08)	
	Extremely Severe	0	0		0		0	
**Anxiety**	Normal	22 (5.5)	47 (1.02)	<0.001	84.37 (12.92)	<0.001	56.21 (7.87)	<0.001
	Mild	40 (10)	46.17 (4.93)		82.26 (10.26)		58.97 (6.89)	
	Moderate	191 (47.75)	44.95 (4.12)		82.30 (8.28)		55.41 (6.93)	
	Severe	141 (35.25)	43.65 (3.94)		78.36 (8.79)		52.75 (7.40)	
	Extremely Severe	6 (1.5)	42.33 (4.17)		76 (7.32)		52.54 (4.37)	
**Stress**	Normal	108 (27)	45.78 (4.48)	0.008	82.80 (9.87)	0.001	56.17 (7.94)	0.002
	Mild	168 (42)	44.29 (4.17)		81.56 (8.52)		55.36 (6.60)	
	Moderate	124 (31)	44.28 (4.12)		78.43 (8.91)		52.95 (7.41)	
	Severe	0	0		0		0	
	Extremely Severe	0	0		0		0	

The results in Table 8 show the correlation between variables. According to the results, there was a negative and significant correlation between MHL and depression (*r* = −0.236), anxiety (*r* = −0.243), and stress (*r* = −0.155) (*P* < 0.001). There was a positive and significant correlation between MHL and D-Lit (*r* = 0.186) (*P* < 0.001). There was a negative and significant correlation between D-Lit and depression (*r* = −0.192), anxiety (*r* = −0.238), and stress (*r* = −0.156) (*P* < 0.001). There was a positive and significant correlation between the ability to recognize disorders (*r* = 0.163) and knowledge of self-treatment (*r* = 0.154) with DQOL (P <0.001). There was a negative and significant correlation between the state of depression (*r* = −0.251), anxiety (*r* = −0.257), stress (*r* = −0.203), and DQOL (*P* < 0.001) (Table 8).

**Table 8:** Pearson correlation between the variables.

**Variables**	**a**.	**b**.	**c**.	**d**.	**e**.	**f**.	**g**.	**h**.	**i**.	**j**.	**k**.	**l**.	**m**.	**n**.	**o**.	**p**.
a. Ability to recognize disorders	1	.														
b. Knowledge of risk factors and causes	0.136^**^	1														
c. knowledge of self-treatment	0.167^**^	0.034	1													
d. Knowledge of the professional help available	0.029	−0.056	0.184^**^	1												
e. Knowledge of where to seek information	0.316^**^	0.051	0.110^*^	−0.085	1											
f. Attitudes that promote recognition or appropriate help-seeking behavior	−0.076	−0.107^*^	0.161^**^	0.114^*^	0.031	1										
g. Mental health literacy (MHL)	0.552^**^	0.179^**^	0.389^**^	0.227^**^	0.553^**^	0.655^**^	1									
h. Depression	−0.220^**^	−0.070	−0.194^**^	−0.078	−0.218^**^	−0.010	−0.236^**^	1								
i. Anxiety	−0.220^**^	−0.118^*^	−0.229^**^	−0.064	−0.267^**^	0.025	−0.243^**^	0.729^**^	1							
j. Stress	−0.195^**^	−0.144^**^	−0.099^*^	0.037	−0.272^**^	0.102^*^	−0.155^**^	0.740^**^	0.672^**^	1						
k. Knowledge of the psychological symptoms	0.288^**^	−0.034	0.340^**^	0.248^**^	0.106^*^	0.117^*^	0.320^**^	−0.246^**^	−0.226^**^	−0.127^*^	1					
l. Knowledge about the effectiveness of available treatment methods	−0.128^*^	−0.131^**^	−0.006	0.070	0.047	0.098	0.014	−0.038	−0.022	−0.016	−0.099^*^	1				
m. Knowledge of cognitive-behavioral symptoms	−0.221^**^	0.013	−0.265^**^	−0.237^**^	−0.086	−0.082	−0.252^**^	0.096	0.028	−0.020	−0.372^**^	0.090	1			
n. Knowledge of taking medications and their side effects	0.086	−0.070	0.067	0.112^*^	0.237^**^	0.073	0.191^**^	−0.105^*^	−0.148^**^	−0.078	0.063	0.136^**^	−0.050	1		
o. Knowledge of disease severity	0.183^**^	0.069	0.187^**^	0.191^**^	0.116^*^	0.028	0.209^**^	−0.144^**^	−0.148^**^	−0.089	0.239^**^	−0.080	−0.238^**^	0.168^**^	1	
p. Depression literacy (D- Lit)	0.077	−0.079	0.119^*^	0.128^*^	0.167^**^	0.099^*^	0.186^**^	−0.192^**^	−0.238^**^	−0.156^**^	0.450^**^	0.408^**^	0.360^**^	0.566^**^	0.277^**^	1
q. DQOL	0.163^**^	0.073	0.154^**^	0.092	−0.010	−0.034	0.092	−0.251^**^	−0.257^**^	−0.203^**^	0.303^**^	−0.062	−0.150^**^	−0.035	0.069	0.074

^*^P <0.05, ^**^P <0.001.

## Discussion

This research showed that MHL was not high and that only 5.8% of patients had high D-Lit scores and were able to answer 15 or more questions correctly. However, in this study, only 7.8% of the patients were free of depression, 5.5% had no anxiety, and 27% were not stressed. In a previous study, the results showed that these patients had insufficient health literacy regarding their illness, and the provision of simple and understandable educational resources for the diabetic population was effective in increasing their health literacy (47). HL assessment is an important prerequisite for reinforcing the proper management of DT2, following treatment, and adopting more flexible health policies (48).

In this study, there was a significant and negative correlation between D-Lit and MHL and the levels of depression, anxiety, and stress. Additionally, there was a significant and negative correlation between levels of depression, anxiety, stress, and the QOL. These results indicate that increasing MHL and D-Lit can decrease the prevalence of anxiety, depression, and stress while improving QOL. These results can be useful for designing educational programs aimed at preventing mental illness and improving T2DM patients' quality of life. Other research indicates that people with low HL are more likely to exhibit depressive symptoms or be considered depressed (49, 50). A previous study found that depressive symptoms affect self-care management and quality of care, negatively affecting health outcomes in people with diabetes (51–53). A previous study also showed that COVID-19 profoundly affects anxiety levels, impacting patients' psychological wellbeing (54). A study from China observed that enhancing D-Lit during the pandemic reduced depression, (55) while another study noted that MHL was negatively correlated with stress and positively correlated with psychological health during the COVID-19 pandemic (56). Therefore, adequate MHL can not only support individuals in critical situations but also play a crucial role in preventing mental disorders.

In this study, there was a positive and significant correlation between knowledge of psychological symptoms, the ability to recognize disorders, and QOL. It appears that people's ability to recognize mental disorder symptoms in the early stages helps them take prompt actions, while awareness of available treatment options facilitates their use of appropriate interventions. As a result, these proactive measures can reduce the risk of various complications, thereby enhancing QOL. The findings of several studies have reported a significant relationship between the ability to diagnose mental disorders and the seeking of mental health services (57–59). People who are adept at diagnosing various types of mental disorders are more likely to seek mental health services, allowing for the early diagnosis and appropriate treatment of their conditions (57–59).

This research revealed a statistically significant relationship between age and lower levels of stress and anxiety among people aged 30 years. One study found that early-onset T2DM is associated with mood disorders, anxiety, and stress (60). The findings of another study indicated that younger people exhibit higher levels of depression and anxiety than older people, which is likely due to their low levels of experience in managing the stresses related to treatment and diabetes challenges (61). Additionally, the effect of the disease on physical and mental performance and QOL contribute to higher rates of depression and anxiety among younger patients (61). The findings of our study contrast those reported in the Palizgir study, which indicated different age-related impacts on psychological wellbeing (61). More research is needed to determine whether there is a relationship between age, stress, and anxiety in T2DM. Other factors, such as the stress associated with managing chronic illnesses, may also contribute to stress and anxiety in patients with T2DM.

There was a statistically significant relationship between education level and anxiety, with patients with elementary education exhibiting higher levels of anxiety compared to those with higher academic education, who displayed lower levels of anxiety. The level of education has another protective effect against anxiety, as people with higher education levels tend to use health and treatment services and have no problem finding places that provide such services (62). In addition, higher education levels are associated with a decreased likelihood of engaging in unhealthy behaviors (63, 64). The findings of a previous study showed that a high education level is a protective factor against anxiety and depression (63). Another study found that patients with T2DM who had low education levels were more likely to experience anxiety (65).

There was also a significant relationship between education levels and knowledge of where to find information, with those with higher education levels having higher knowledge. In line with this result, a relationship between education and HL has already been reported (66). We found a positive relationship between education levels and HL scores, as in other studies (66–68). More educated people have fewer problems finding valid health care and understanding health information.

There was a significant relationship between economic status and MHL; patients with better economic conditions had higher levels of MHL. These results are similar to those of a previous study (69), suggesting that socioeconomic status may be an independent variable that can affect HL directly through education and indirectly through access to digital medical content using tablets and smartphones. In addition, this study found a significant relationship between receiving mental health information and the levels of MHL and D-Lit, and those who received mental health information had higher MHL.

The results of this study can be combined and explained with the mind sponge theory, which was first presented by Vuong and Napier (70). According to the mind-sponge model, the human mind acts like a sponge, absorbing values and information that are compatible with one's mindset and discarding those that are not. This metaphorical sponge consists of several layers, with the mindset encompassing an individual's core values positioned at the center. Information, values, cultural norms, and environmental beliefs are evaluated based on their alignment with these core values, determining their acceptance or rejection (71–74).

According to Jorm's definition of MHL, correct knowledge, beliefs, and attitudes about identifying mental illnesses, their causes, risk factors, and avenues for seeking help are the fundamental components of MHL (75). As a result, by implementing strategies and policies to promote MHL, it can be expected that societal mindsets will evolve over time, leading to the adoption of more correct values, beliefs, and attitudes toward mental issues, replacing erroneous ones. Once the core values concerning mental health issues are corrected. people will be better equipped to judge and use the information around them. More correct information will be absorbed by the “mind sponge,” while incorrect information will be rejected. This shift lays the groundwork for adopting correct behaviors, such as seeking professional help when in need. For example, a person experiencing symptoms of depression but possessing a high level of MHL due to correct knowledge, attitudes, and values, is likely to make more accurate cost-benefit analyses. Consequently, while absorbing more accurate information from the environment, such people are more inclined to seek professional help and adopt self-help measures. Conversely, in individuals with low MHL and incorrect values, the absorption of incorrect information, such as methods for suicide attempts or the avoidance of psychological help due to stigmatizing attitudes, can be expected.

As a result, improving MHL, specifically depression health literacy, can facilitate the restructuring of the knowledge, beliefs, values, and attitudes in the mind-sponge mindset of people with diabetes, leading to more correct judgments and, ultimately, more correct help-seeking behaviors. Consequently, the occurrence of mental disorders, such as depression and anxiety, is expected to be prevented. If these disorders occur, the patient is more likely to receive timely treatment and ultimately experience a better quality of life.

Quality of life plays a fundamental role in achieving treatment goals and the early diagnosis of diabetes (76). The significant prevalence of depression, stress, and anxiety among people with diabetes and their potential negative impact on quality of life are concerning. The findings of this study suggest that screening and initial assessment of individuals with diabetes at the community-based care level are necessary to address their mental health problems. As a result, it is advisable for primary healthcare providers to regularly evaluate patients' mental health while implementing routine interventions, such as monitoring patients' glycemic status, dietary compliance, and activity levels and referring patients to mental health specialists when necessary. Improving mental health and depression health literacy can form the cornerstone of mental health education for all people with diabetes. Educators, armed with a thorough understanding of the main components of MHL and depression literacy, can provide more targeted, coherent, and useful education, ultimately leading to transformative changes in the values and mindsets of patients. Depression is also associated with an increased risk of hospitalization (for any reason) in patients with diabetes (77). Therefore, nurses and practitioners should not neglect to take the mental history of patients with diabetes into account in addition to initial clinical evaluations. Finally, depression and anxiety contribute to increased healthcare system costs (78), highlighting the need for policymakers to invest in preventive interventions, especially in improving MHL and depression literacy.

This study has limitations that should be considered when interpreting the findings. The cross-sectional nature of this study restricts our ability to establish causal relationships among the variables. Although we observed associations between MHL, psychological status (anxiety, depression, and stress), and quality of life, we cannot definitively conclude that one variable causes the other. Longitudinal studies are required to further investigate the temporal relationships between these variables. In addition, the sample of 400 patients with DT2 from Gonabad, Iran, may not be representative of the entire diabetic population. Consequently, these findings may not be generalizable to patients in other geographic areas or those with different demographic characteristics. Participants' recall accuracy and the potential for socially desirable responses could also skew results. Objective measures, such as clinical assessments, can help address these concerns. The DASS-21 questionnaire, while useful, is used to assess depression, anxiety, and stress; a more comprehensive assessment using in-depth clinical interviews or specialized mental health questionnaires can provide a more accurate understanding of the participants' mental health status.

## Conclusion

Our study revealed that lower MHL and D-Lit scores among patients with T2DM were associated with higher levels of anxiety, depression, and stress, as well as a lower quality of life. These findings demonstrate the important role of MHL and D-Lit in promoting psychological wellbeing and overall quality of life in this population. The findings showed that the MHL and D-Lit levels in patients with T2DM were inadequate. Considering the high risk of various mental disorders in patients with T2DM, it is necessary to improve their MHL to detect such disorders in the early stages, seek mental health services, and receive available treatments. In addition, designing preventive programs to improve the mental health of patients with T2DM can prevent the occurrence of mental disorders and improve their QOL. For example, the PATIENT strategy (P: patient's perception; A: assessment; T: tailored approach; I: iterative evaluation; E: education; N: non-pharmacological approach; T: team) used in the previous study can be applied (79).

## Data availability statement

The original contributions presented in the study are included in the article/Supplementary material, further inquiries can be directed to the corresponding author.

## Ethics statement

The studies involving humans were approved by Ethics Committee of Gonabad University of Medical Sciences with the code of ethics IR.GMU.REC.1401.017. The studies were conducted in accordance with the local legislation and institutional requirements. The participants provided their written informed consent to participate in this study.

## Author contributions

AJ: Conceptualization, Data curation, Formal analysis, Investigation, Methodology, Project administration, Software, Supervision, Validation, Writing – original draft, Writing – review & editing. MM: Conceptualization, Investigation, Project administration, Supervision, Writing – original draft, Writing – review & editing. FN: Conceptualization, Investigation, Project administration, Writing – original draft, Writing – review & editing. MG-G: Conceptualization, Investigation, Software, Writing – original draft, Writing – review & editing. VA: Conceptualization, Investigation, Writing – original draft, Writing – review & editing. KK: Conceptualization, Investigation, Writing – original draft, Writing – review & editing. MN: Conceptualization, Investigation, Methodology, Project administration, Software, Supervision, Writing – original draft, Writing – review & editing.
