# Supplementary material for: Depression literacy, mental health literacy, and their relationship with psychological status and quality of life in patients with type 2 diabetes mellitus
Source: Front Public Health. 2024 Jul 11;12:1421053. doi: 10.3389/fpubh.2024.1421053 (PMC11269263; doi:10.3389/fpubh.2024.1421053)
Supplement: Supplementary file 1 [file Table_1.docx]

**Table S1.** Results of Tukey's post hoc between demographic factors and depression

| **Variables** | | | Mean Difference (I-J) | Std. Error | | Sig. | | 95% Confidence Interval | | | |
| --- | --- | --- | --- | --- | --- | --- | --- | --- | --- | --- | --- |
|  |  |  |  |  |  |  |  | Lower Bound | | Upper Bound | |
| **Age group** | <30 | 30-50 | -1.02511 | | .90468 | | .494 | | -3.1534 | | 1.1032 |
|  |  | >50 | -1.82947 | | .92325 | | .118 | | -4.0015 | | .3425 |
|  | 30-50 | <30 | 1.02511 | | .90468 | | .494 | | -1.1032 | | 3.1534 |
|  |  | >50 | -.80436 | | .40674 | | .119 | | -1.7612 | | .1525 |
|  | >50 | <30 | 1.82947 | | .92325 | | .118 | | -.3425 | | 4.0015 |
|  |  | 30-50 | .80436 | | .40674 | | .119 | | -.1525 | | 1.7612 |
| **Education level** | Illiteracy | Elementary | -1.35096 | | 1.26263 | | .893 | | -4.9674 | | 2.2655 |
|  |  | Middle school | -1.62937 | | 1.34298 | | .830 | | -5.4759 | | 2.2172 |
|  |  | High school | .23077 | | 1.30404 | | 1.000 | | -3.5043 | | 3.9658 |
|  |  | Diploma | -1.53846 | | 1.12382 | | .746 | | -4.7573 | | 1.6804 |
|  |  | Academic | -.74725 | | 1.10212 | | .984 | | -3.9039 | | 2.4094 |
|  | Elementary | Illiteracy | 1.35096 | | 1.26263 | | .893 | | -2.2655 | | 4.9674 |
|  |  | Middle school | -.27841 | | 1.06323 | | 1.000 | | -3.3237 | | 2.7669 |
|  |  | High school | 1.58173 | | 1.01361 | | .625 | | -1.3215 | | 4.4849 |
|  |  | Diploma | -.18750 | | .76801 | | 1.000 | | -2.3872 | | 2.0122 |
|  |  | Academic | .60371 | | .73589 | | .964 | | -1.5040 | | 2.7115 |
|  | Middle school | Illiteracy | 1.62937 | | 1.34298 | | .830 | | -2.2172 | | 5.4759 |
|  |  | Elementary | .27841 | | 1.06323 | | 1.000 | | -2.7669 | | 3.3237 |
|  |  | High school | 1.86014 | | 1.11209 | | .551 | | -1.3251 | | 5.0454 |
|  |  | Diploma | .09091 | | .89397 | | 1.000 | | -2.4696 | | 2.6514 |
|  |  | Academic | .88212 | | .86653 | | .912 | | -1.5998 | | 3.3641 |
|  | High school | Illiteracy | -.23077 | | 1.30404 | | 1.000 | | -3.9658 | | 3.5043 |
|  |  | Elementary | -1.58173 | | 1.01361 | | .625 | | -4.4849 | | 1.3215 |
|  |  | Middle school | -1.86014 | | 1.11209 | | .551 | | -5.0454 | | 1.3251 |
|  |  | Diploma | -1.76923 | | .83434 | | .279 | | -4.1589 | | .6205 |
|  |  | Academic | -.97802 | | .80487 | | .829 | | -3.2833 | | 1.3273 |
|  | Diploma | Illiteracy | 1.53846 | | 1.12382 | | .746 | | -1.6804 | | 4.7573 |
|  |  | Elementary | .18750 | | .76801 | | 1.000 | | -2.0122 | | 2.3872 |
|  |  | Middle school | -.09091 | | .89397 | | 1.000 | | -2.6514 | | 2.4696 |
|  |  | High school | 1.76923 | | .83434 | | .279 | | -.6205 | | 4.1589 |
|  |  | Academic | .79121 | | .45854 | | .516 | | -.5221 | | 2.1046 |
|  | Academic | Illiteracy | .74725 | | 1.10212 | | .984 | | -2.4094 | | 3.9039 |
|  |  | Elementary | -.60371 | | .73589 | | .964 | | -2.7115 | | 1.5040 |
|  |  | Middle school | -.88212 | | .86653 | | .912 | | -3.3641 | | 1.5998 |
|  |  | High school | .97802 | | .80487 | | .829 | | -1.3273 | | 3.2833 |
|  |  | Diploma | -.79121 | | .45854 | | .516 | | -2.1046 | | .5221 |
| **Job** | Housewife | Employed | .17506 | | .56907 | | .998 | | -1.3847 | | 1.7348 |
|  |  | Retired | .04434 | | .66608 | | 1.000 | | -1.7813 | | 1.8700 |
|  |  | Self-employed | 1.08469 | | .55906 | | .298 | | -.4476 | | 2.6170 |
|  |  | Labor | .78363 | | .77914 | | .853 | | -1.3518 | | 2.9191 |
|  | Employed | Housewife | -.17506 | | .56907 | | .998 | | -1.7348 | | 1.3847 |
|  |  | Retired | -.13071 | | .65178 | | 1.000 | | -1.9171 | | 1.6557 |
|  |  | Self-employed | .90963 | | .54194 | | .449 | | -.5757 | | 2.3950 |
|  |  | Labor | .60857 | | .76695 | | .932 | | -1.4935 | | 2.7106 |
|  | Retired | Housewife | -.04434 | | .66608 | | 1.000 | | -1.8700 | | 1.7813 |
|  |  | Employed | .13071 | | .65178 | | 1.000 | | -1.6557 | | 1.9171 |
|  |  | Self-employed | 1.04034 | | .64306 | | .487 | | -.7222 | | 2.8028 |
|  |  | Labor | .73929 | | .84144 | | .905 | | -1.5670 | | 3.0455 |
|  | Self-employed | Housewife | -1.08469 | | .55906 | | .298 | | -2.6170 | | .4476 |
|  |  | Employed | -.90963 | | .54194 | | .449 | | -2.3950 | | .5757 |
|  |  | Retired | -1.04034 | | .64306 | | .487 | | -2.8028 | | .7222 |
|  |  | Labor | -.30106 | | .75955 | | .995 | | -2.3828 | | 1.7807 |
|  | labor | Housewife | -.78363 | | .77914 | | .853 | | -2.9191 | | 1.3518 |
|  |  | Employed | -.60857 | | .76695 | | .932 | | -2.7106 | | 1.4935 |
|  |  | Retired | -.73929 | | .84144 | | .905 | | -3.0455 | | 1.5670 |
|  |  | Self-employed | .30106 | | .75955 | | .995 | | -1.7807 | | 2.3828 |
| **Duration of diabetes** | ≤ 5 | 6-10 | -.40485 | | .49770 | | .695 | | -1.5762 | | .7665 |
|  |  | >10 | -1.30924^*^ | | .50564 | | .027 | | -2.4993 | | -.1192 |
|  | 6-10 | ≤ 5 | .40485 | | .49770 | | .695 | | -.7665 | | 1.5762 |
|  |  | >10 | -.90439 | | .56022 | | .241 | | -2.2229 | | .4141 |
|  | >10 | ≤ 5 | 1.30924^*^ | | .50564 | | .027 | | .1192 | | 2.4993 |
|  |  | 6-10 | .90439 | | .56022 | | .241 | | -.4141 | | 2.2229 |
| **Method of obtaining health information** | Physician/ Health care providers | Internet | .41670 | | .54341 | | .988 | | -1.1939 | | 2.0273 |
|  |  | Newspapers/magazines | -2.02426 | | 1.03139 | | .440 | | -5.0811 | | 1.0326 |
|  |  | Friends and acquaintances | -.53703 | | .68399 | | .986 | | -2.5642 | | 1.4902 |
|  |  | Book | -.41250 | | 1.08662 | | 1.000 | | -3.6330 | | 2.8080 |
|  |  | Radio, television and satellite | -.71250 | | .61063 | | .906 | | -2.5223 | | 1.0973 |
|  |  | I dont Know | .58750 | | 1.08662 | | .998 | | -2.6330 | | 3.8080 |
|  | Internet | Physician/ Health care providers | -.41670 | | .54341 | | .988 | | -2.0273 | | 1.1939 |
|  |  | Newspapers/magazines | -2.44096 | | .99307 | | .178 | | -5.3842 | | .5023 |
|  |  | Friends and acquaintances | -.95373 | | .62472 | | .729 | | -2.8053 | | .8978 |
|  |  | Book | -.82920 | | 1.05032 | | .986 | | -3.9421 | | 2.2837 |
|  |  | Radio, television and satellite | -1.12920 | | .54341 | | .368 | | -2.7398 | | .4814 |
|  |  | I dont Know | .17080 | | 1.05032 | | 1.000 | | -2.9421 | | 3.2837 |
|  | Newspapers/ magazines | Physician/ Health care providers | 2.02426 | | 1.03139 | | .440 | | -1.0326 | | 5.0811 |
|  |  | Internet | 2.44096 | | .99307 | | .178 | | -.5023 | | 5.3842 |
|  |  | Friends and acquaintances | 1.48724 | | 1.07645 | | .811 | | -1.7031 | | 4.6776 |
|  |  | Book | 1.61176 | | 1.36808 | | .902 | | -2.4429 | | 5.6665 |
|  |  | Radio, television and satellite | 1.31176 | | 1.03139 | | .864 | | -1.7451 | | 4.3686 |
|  |  | I don’t Know | 2.61176 | | 1.36808 | | .475 | | -1.4429 | | 6.6665 |
|  | Friends and acquaintances | Physician/ Health care providers | .53703 | | .68399 | | .986 | | -1.4902 | | 2.5642 |
|  |  | Internet | .95373 | | .62472 | | .729 | | -.8978 | | 2.8053 |
|  |  | Newspapers/magazines | -1.48724 | | 1.07645 | | .811 | | -4.6776 | | 1.7031 |
|  |  | Book | .12453 | | 1.12947 | | 1.000 | | -3.2230 | | 3.4721 |
|  |  | Radio, television and satellite | -.17547 | | .68399 | | 1.000 | | -2.2027 | | 1.8517 |
|  |  | I dont Know | 1.12453 | | 1.12947 | | .955 | | -2.2230 | | 4.4721 |
|  | Book | Physician/ Health care providers | .41250 | | 1.08662 | | 1.000 | | -2.8080 | | 3.6330 |
|  |  | Internet | .82920 | | 1.05032 | | .986 | | -2.2837 | | 3.9421 |
|  |  | Newspapers/magazines | -1.61176 | | 1.36808 | | .902 | | -5.6665 | | 2.4429 |
|  |  | Friends and acquaintances | -.12453 | | 1.12947 | | 1.000 | | -3.4721 | | 3.2230 |
|  |  | Radio, television and satellite | -.30000 | | 1.08662 | | 1.000 | | -3.5205 | | 2.9205 |
|  |  | I dont Know | 1.00000 | | 1.41018 | | .992 | | -3.1795 | | 5.1795 |
|  | Radio, television and satellite | Physician/ Health care providers | .71250 | | .61063 | | .906 | | -1.0973 | | 2.5223 |
|  |  | Internet | 1.12920 | | .54341 | | .368 | | -.4814 | | 2.7398 |
|  |  | Newspapers/magazines | -1.31176 | | 1.03139 | | .864 | | -4.3686 | | 1.7451 |
|  |  | Friends and acquaintances | .17547 | | .68399 | | 1.000 | | -1.8517 | | 2.2027 |
|  |  | Book | .30000 | | 1.08662 | | 1.000 | | -2.9205 | | 3.5205 |
|  |  | I dont Know | 1.30000 | | 1.08662 | | .895 | | -1.9205 | | 4.5205 |
|  | I do not know | Physician/ Health care providers | -.58750 | | 1.08662 | | .998 | | -3.8080 | | 2.6330 |
|  |  | Internet | -.17080 | | 1.05032 | | 1.000 | | -3.2837 | | 2.9421 |
|  |  | Newspapers/magazines | -2.61176 | | 1.36808 | | .475 | | -6.6665 | | 1.4429 |
|  |  | Friends and acquaintances | -1.12453 | | 1.12947 | | .955 | | -4.4721 | | 2.2230 |
|  |  | Book | -1.00000 | | 1.41018 | | .992 | | -5.1795 | | 3.1795 |
|  |  | Radio, television and satellite | -1.30000 | | 1.08662 | | .895 | | -4.5205 | | 1.9205 |
| **Method of obtaining information related to mental illness** | Physician/ Health care providers | Psychologist/Psychiatrist | -1.14526 | | .98984 | | .857 | | -3.9842 | | 1.6936 |
|  |  | Friends and acquaintances | -.46857 | | .78893 | | .991 | | -2.7313 | | 1.7941 |
|  |  | Book | -1.70667 | | 1.35955 | | .809 | | -5.6059 | | 2.1926 |
|  |  | Internet | -.39659 | | .55962 | | .981 | | -2.0016 | | 1.2084 |
|  |  | Radio, television and satellite, TV | -1.80190 | | .74275 | | .151 | | -3.9322 | | .3284 |
|  | Psychologist/Psychiatrist | Physician/ Health care providers | 1.14526 | | .98984 | | .857 | | -1.6936 | | 3.9842 |
|  |  | Friends and acquaintances | .67669 | | 1.09823 | | .990 | | -2.4731 | | 3.8265 |
|  |  | Book | -.56140 | | 1.55951 | | .999 | | -5.0342 | | 3.9113 |
|  |  | Internet | .74867 | | .94704 | | .969 | | -1.9675 | | 3.4648 |
|  |  | Radio, television and satellite, TV | -.65664 | | 1.06554 | | .990 | | -3.7127 | | 2.3994 |
|  | Friends and acquaintances | Physician/ Health care providers | .46857 | | .78893 | | .991 | | -1.7941 | | 2.7313 |
|  |  | Psychologist/Psychiatrist | -.67669 | | 1.09823 | | .990 | | -3.8265 | | 2.4731 |
|  |  | Book | -1.23810 | | 1.44038 | | .956 | | -5.3692 | | 2.8930 |
|  |  | Internet | .07198 | | .73451 | | 1.000 | | -2.0346 | | 2.1786 |
|  |  | Radio, television and satellite, TV | -1.33333 | | .88205 | | .657 | | -3.8631 | | 1.1964 |
|  | Book | Physician/ Health care providers | 1.70667 | | 1.35955 | | .809 | | -2.1926 | | 5.6059 |
|  |  | Psychologist/Psychiatrist | .56140 | | 1.55951 | | .999 | | -3.9113 | | 5.0342 |
|  |  | Friends and acquaintances | 1.23810 | | 1.44038 | | .956 | | -2.8930 | | 5.3692 |
|  |  | Internet | 1.31008 | | 1.32871 | | .922 | | -2.5007 | | 5.1209 |
|  |  | Radio, television and satellite, TV | -.09524 | | 1.41562 | | 1.000 | | -4.1553 | | 3.9648 |
|  | Internet | Physician/ Health care providers | .39659 | | .55962 | | .981 | | -1.2084 | | 2.0016 |
|  |  | Psychologist/Psychiatrist | -.74867 | | .94704 | | .969 | | -3.4648 | | 1.9675 |
|  |  | Friends and acquaintances | -.07198 | | .73451 | | 1.000 | | -2.1786 | | 2.0346 |
|  |  | Book | -1.31008 | | 1.32871 | | .922 | | -5.1209 | | 2.5007 |
|  |  | Radio, television and satellite, TV | -1.40532 | | .68468 | | .315 | | -3.3690 | | .5584 |
|  | Radio, television and satellite | Physician/ Health care providers | 1.80190 | | .74275 | | .151 | | -.3284 | | 3.9322 |
|  |  | Psychologist/Psychiatrist | .65664 | | 1.06554 | | .990 | | -2.3994 | | 3.7127 |
|  |  | Friends and acquaintances | 1.33333 | | .88205 | | .657 | | -1.1964 | | 3.8631 |
|  |  | Book | .09524 | | 1.41562 | | 1.000 | | -3.9648 | | 4.1553 |
|  |  | Internet | 1.40532 | | .68468 | | .315 | | -.5584 | | 3.3690 |
| *. The mean difference is significant at the 0.05 level. | | | | | | | | | | | |
