# Supplementary material for: Depression literacy, mental health literacy, and their relationship with psychological status and quality of life in patients with type 2 diabetes mellitus
Source: Front Public Health. 2024 Jul 11;12:1421053. doi: 10.3389/fpubh.2024.1421053 (PMC11269263; doi:10.3389/fpubh.2024.1421053)
Supplement: Supplementary file 2 [file Table_2.docx]

**Table S2.** Results of Tukey's post hoc between demographic factors and anxiety

| **Variables** | | | Mean Difference (I-J) | Std. Error | Sig. | 95% Confidence Interval | |  |
| --- | --- | --- | --- | --- | --- | --- | --- | --- |
|  |  |  |  |  |  | Lower Bound | Upper Bound |  |
| **Age group** | <30 | 30-50 | -1.28559 | .77373 | .221 | -3.1058 | .5346 | |
|  |  | >50 | -2.73245^*^ | .78961 | .002 | -4.5900 | -.8749 | |
|  | 30-50 | <30 | 1.28559 | .77373 | .221 | -.5346 | 3.1058 | |
|  |  | >50 | -1.44686^*^ | .34786 | .000 | -2.2652 | -.6285 | |
|  | >50 | <30 | 2.73245^*^ | .78961 | .002 | .8749 | 4.5900 | |
|  |  | 30-50 | 1.44686^*^ | .34786 | .000 | .6285 | 2.2652 | |
| **Education level** | Illiteracy | Elementary | -1.16827 | 1.08525 | .891 | -4.2766 | 1.9401 | |
|  |  | Middle school | -.32168 | 1.15431 | 1.000 | -3.6279 | 2.9845 | |
|  |  | High school | 1.07692 | 1.12084 | .930 | -2.1334 | 4.2872 | |
|  |  | Diploma | .44467 | .96593 | .997 | -2.3220 | 3.2113 | |
|  |  | Academic | 1.26374 | .94728 | .766 | -1.4495 | 3.9770 | |
|  | Elementary | Illiteracy | 1.16827 | 1.08525 | .891 | -1.9401 | 4.2766 | |
|  |  | Middle school | .84659 | .91386 | .940 | -1.7709 | 3.4641 | |
|  |  | High school | 2.24519 | .87121 | .105 | -.2501 | 4.7405 | |
|  |  | Diploma | 1.61294 | .66011 | .144 | -.2778 | 3.5036 | |
|  |  | Academic | 2.43201^*^ | .63251 | .002 | .6204 | 4.2436 | |
|  | Middle school | Illiteracy | .32168 | 1.15431 | 1.000 | -2.9845 | 3.6279 | |
|  |  | Elementary | -.84659 | .91386 | .940 | -3.4641 | 1.7709 | |
|  |  | High school | 1.39860 | .95586 | .688 | -1.3392 | 4.1364 | |
|  |  | Diploma | .76635 | .76838 | .919 | -1.4345 | 2.9671 | |
|  |  | Academic | 1.58541 | .74480 | .275 | -.5478 | 3.7187 | |
|  | High school | Illiteracy | -1.07692 | 1.12084 | .930 | -4.2872 | 2.1334 | |
|  |  | Elementary | -2.24519 | .87121 | .105 | -4.7405 | .2501 | |
|  |  | Middle school | -1.39860 | .95586 | .688 | -4.1364 | 1.3392 | |
|  |  | Diploma | -.63225 | .71713 | .951 | -2.6862 | 1.4217 | |
|  |  | Academic | .18681 | .69180 | 1.000 | -1.7946 | 2.1683 | |
|  | Diploma | Illiteracy | -.44467 | .96593 | .997 | -3.2113 | 2.3220 | |
|  |  | Elementary | -1.61294 | .66011 | .144 | -3.5036 | .2778 | |
|  |  | Middle school | -.76635 | .76838 | .919 | -2.9671 | 1.4345 | |
|  |  | High school | .63225 | .71713 | .951 | -1.4217 | 2.6862 | |
|  |  | Academic | .81907 | .39412 | .301 | -.3098 | 1.9479 | |
|  | Academic | Illiteracy | -1.26374 | .94728 | .766 | -3.9770 | 1.4495 | |
|  |  | Elementary | -2.43201^*^ | .63251 | .002 | -4.2436 | -.6204 | |
|  |  | Middle school | -1.58541 | .74480 | .275 | -3.7187 | .5478 | |
|  |  | High school | -.18681 | .69180 | 1.000 | -2.1683 | 1.7946 | |
|  |  | Diploma | -.81907 | .39412 | .301 | -1.9479 | .3098 | |
| **Job** | Housewife | Employed | .25101 | .48440 | .986 | -1.0766 | 1.5787 | |
|  |  | Retired | -1.34470 | .56697 | .125 | -2.8987 | .2093 | |
|  |  | Self-employed | .37620 | .47588 | .933 | -.9281 | 1.6805 | |
|  |  | Labor | .33387 | .66321 | .987 | -1.4839 | 2.1516 | |
|  | Employed | Housewife | -.25101 | .48440 | .986 | -1.5787 | 1.0766 | |
|  |  | Retired | -1.59571^*^ | .55480 | .034 | -3.1163 | -.0751 | |
|  |  | Self-employed | .12519 | .46131 | .999 | -1.1392 | 1.3895 | |
|  |  | Labor | .08286 | .65283 | 1.000 | -1.7064 | 1.8722 | |
|  | Retired | Housewife | 1.34470 | .56697 | .125 | -.2093 | 2.8987 | |
|  |  | Employed | 1.59571^*^ | .55480 | .034 | .0751 | 3.1163 | |
|  |  | Self-employed | 1.72090^*^ | .54737 | .015 | .2206 | 3.2212 | |
|  |  | Labor | 1.67857 | .71624 | .134 | -.2845 | 3.6417 | |
|  | Self-employed | Housewife | -.37620 | .47588 | .933 | -1.6805 | .9281 | |
|  |  | Employed | -.12519 | .46131 | .999 | -1.3895 | 1.1392 | |
|  |  | Retired | -1.72090^*^ | .54737 | .015 | -3.2212 | -.2206 | |
|  |  | Labor | -.04233 | .64653 | 1.000 | -1.8144 | 1.7297 | |
|  | labor | Housewife | -.33387 | .66321 | .987 | -2.1516 | 1.4839 | |
|  |  | Employed | -.08286 | .65283 | 1.000 | -1.8722 | 1.7064 | |
|  |  | Retired | -1.67857 | .71624 | .134 | -3.6417 | .2845 | |
|  |  | Self-employed | .04233 | .64653 | 1.000 | -1.7297 | 1.8144 | |
| **Duration of diabetes** | ≤ 5 | 6-10 | -.09354 | .41803 | .973 | -1.0774 | .8903 | |
|  |  | >10 | -1.90037^*^ | .42470 | .000 | -2.8999 | -.9008 | |
|  | 6-10 | ≤ 5 | .09354 | .41803 | .973 | -.8903 | 1.0774 | |
|  |  | >10 | -1.80683^*^ | .47054 | .000 | -2.9143 | -.6994 | |
|  | >10 | ≤ 5 | 1.90037^*^ | .42470 | .000 | .9008 | 2.8999 | |
|  |  | 6-10 | 1.80683^*^ | .47054 | .000 | .6994 | 2.9143 | |
|  | Physician/ Health care providers | Internet | 1.48422^*^ | .46731 | .027 | .0992 | 2.8692 | |
| **Method of obtaining health information** |  | Newspapers/magazines | -1.36250 | .88695 | .723 | -3.9912 | 1.2662 | |
|  |  | Friends and acquaintances | .37335 | .58820 | .996 | -1.3700 | 2.1167 | |
|  |  | Book | .70417 | .93444 | .989 | -2.0653 | 3.4737 | |
|  |  | Radio, television and satellite | -.48750 | .52511 | .968 | -2.0438 | 1.0688 | |
|  |  | I don’t Know | 1.30417 | .93444 | .804 | -1.4653 | 4.0737 | |
|  | Internet | Physician/ Health care providers | -1.48422^*^ | .46731 | .027 | -2.8692 | -.0992 | |
|  |  | Newspapers/magazines | -2.84672^*^ | .85400 | .016 | -5.3778 | -.3156 | |
|  |  | Friends and acquaintances | -1.11087 | .53723 | .374 | -2.7031 | .4814 | |
|  |  | Book | -.78005 | .90323 | .978 | -3.4570 | 1.8969 | |
|  |  | Radio, television and satellite | -1.97172^*^ | .46731 | .001 | -3.3567 | -.5867 | |
|  |  | I dont Know | -.18005 | .90323 | 1.000 | -2.8570 | 2.4969 | |
|  | Newspapers/ magazines | Physician/ Health care providers | 1.36250 | .88695 | .723 | -1.2662 | 3.9912 | |
|  |  | Internet | 2.84672^*^ | .85400 | .016 | .3156 | 5.3778 | |
|  |  | Friends and acquaintances | 1.73585 | .92570 | .498 | -1.0077 | 4.4794 | |
|  |  | Book | 2.06667 | 1.17649 | .578 | -1.4202 | 5.5535 | |
|  |  | Radio, television and satellite | .87500 | .88695 | .957 | -1.7537 | 3.5037 | |
|  |  | I dont Know | 2.66667 | 1.17649 | .263 | -.8202 | 6.1535 | |
|  | Friends and acquaintances | Physician/ Health care providers | -.37335 | .58820 | .996 | -2.1167 | 1.3700 | |
|  |  | Internet | 1.11087 | .53723 | .374 | -.4814 | 2.7031 | |
|  |  | Newspapers/magazines | -1.73585 | .92570 | .498 | -4.4794 | 1.0077 | |
|  |  | Book | .33082 | .97130 | 1.000 | -2.5479 | 3.2096 | |
|  |  | Radio, television and satellite | -.86085 | .58820 | .766 | -2.6042 | .8825 | |
|  |  | I dont Know | .93082 | .97130 | .962 | -1.9479 | 3.8096 | |
|  | Book | Physician/ Health care providers | -.70417 | .93444 | .989 | -3.4737 | 2.0653 | |
|  |  | Internet | .78005 | .90323 | .978 | -1.8969 | 3.4570 | |
|  |  | Newspapers/magazines | -2.06667 | 1.17649 | .578 | -5.5535 | 1.4202 | |
|  |  | Friends and acquaintances | -.33082 | .97130 | 1.000 | -3.2096 | 2.5479 | |
|  |  | Radio, television and satellite | -1.19167 | .93444 | .863 | -3.9612 | 1.5778 | |
|  |  | I dont Know | .60000 | 1.21269 | .999 | -2.9942 | 4.1942 | |
|  | Radio, television and satellite | Physician/ Health care providers | .48750 | .52511 | .968 | -1.0688 | 2.0438 | |
|  |  | Internet | 1.97172^*^ | .46731 | .001 | .5867 | 3.3567 | |
|  |  | Newspapers/magazines | -.87500 | .88695 | .957 | -3.5037 | 1.7537 | |
|  |  | Friends and acquaintances | .86085 | .58820 | .766 | -.8825 | 2.6042 | |
|  |  | Book | 1.19167 | .93444 | .863 | -1.5778 | 3.9612 | |
|  |  | I dont Know | 1.79167 | .93444 | .470 | -.9778 | 4.5612 | |
|  | I do not know | Physician/ Health care providers | -1.30417 | .93444 | .804 | -4.0737 | 1.4653 | |
|  |  | Internet | .18005 | .90323 | 1.000 | -2.4969 | 2.8570 | |
|  |  | Newspapers/magazines | -2.66667 | 1.17649 | .263 | -6.1535 | .8202 | |
|  |  | Friends and acquaintances | -.93082 | .97130 | .962 | -3.8096 | 1.9479 | |
|  |  | Book | -.60000 | 1.21269 | .999 | -4.1942 | 2.9942 | |
|  |  | Radio, television and satellite | -1.79167 | .93444 | .470 | -4.5612 | .9778 | |
| **Method of obtaining information related to mental illness** | Physician/ Health care providers | Psychologist/Psychiatrist | -.05544 | .86376 | 1.000 | -2.5327 | 2.4219 | |
|  |  | Friends and acquaintances | 1.67238 | .68844 | .150 | -.3021 | 3.6469 | |
|  |  | Book | .67556 | 1.18638 | .993 | -2.7270 | 4.0781 | |
|  |  | Internet | 1.22853 | .48834 | .123 | -.1721 | 2.6291 | |
|  |  | Radio, television and satellite, TV | -.97524 | .64814 | .662 | -2.8342 | .8837 | |
|  | Psychologist/Psychiatrist | Physician/ Health care providers | .05544 | .86376 | 1.000 | -2.4219 | 2.5327 | |
|  |  | Friends and acquaintances | 1.72782 | .95834 | .465 | -1.0208 | 4.4764 | |
|  |  | Book | .73099 | 1.36087 | .995 | -3.1720 | 4.6340 | |
|  |  | Internet | 1.28397 | .82641 | .630 | -1.0862 | 3.6541 | |
|  |  | Radio, television and satellite, TV | -.91980 | .92982 | .921 | -3.5866 | 1.7470 | |
|  | Friends and acquaintances | Physician/ Health care providers | -1.67238 | .68844 | .150 | -3.6469 | .3021 | |
|  |  | Psychologist/Psychiatrist | -1.72782 | .95834 | .465 | -4.4764 | 1.0208 | |
|  |  | Book | -.99683 | 1.25691 | .969 | -4.6017 | 2.6081 | |
|  |  | Internet | -.44385 | .64095 | .983 | -2.2821 | 1.3944 | |
|  |  | Radio, television and satellite, TV | -2.64762^*^ | .76970 | .009 | -4.8552 | -.4401 | |
|  | Book | Physician/ Health care providers | -.67556 | 1.18638 | .993 | -4.0781 | 2.7270 | |
|  |  | Psychologist/Psychiatrist | -.73099 | 1.36087 | .995 | -4.6340 | 3.1720 | |
|  |  | Friends and acquaintances | .99683 | 1.25691 | .969 | -2.6081 | 4.6017 | |
|  |  | Internet | .55297 | 1.15947 | .997 | -2.7724 | 3.8784 | |
|  |  | Radio, television and satellite, TV | -1.65079 | 1.23530 | .765 | -5.1937 | 1.8921 | |
|  | Internet | Physician/ Health care providers | -1.22853 | .48834 | .123 | -2.6291 | .1721 | |
|  |  | Psychologist/Psychiatrist | -1.28397 | .82641 | .630 | -3.6541 | 1.0862 | |
|  |  | Friends and acquaintances | .44385 | .64095 | .983 | -1.3944 | 2.2821 | |
|  |  | Book | -.55297 | 1.15947 | .997 | -3.8784 | 2.7724 | |
|  |  | Radio, television and satellite, TV | -2.20377^*^ | .59747 | .004 | -3.9173 | -.4902 | |
|  | Radio, television and satellite | Physician/ Health care providers | .97524 | .64814 | .662 | -.8837 | 2.8342 | |
|  |  | Psychologist/Psychiatrist | .91980 | .92982 | .921 | -1.7470 | 3.5866 | |
|  |  | Friends and acquaintances | 2.64762^*^ | .76970 | .009 | .4401 | 4.8552 | |
|  |  | Book | 1.65079 | 1.23530 | .765 | -1.8921 | 5.1937 | |
|  |  | Internet | 2.20377^*^ | .59747 | .004 | .4902 | 3.9173 | |
| *. The mean difference is significant at the 0.05 level. | | | | | | | |  |
