# Supplementary material for: Depression literacy, mental health literacy, and their relationship with psychological status and quality of life in patients with type 2 diabetes mellitus
Source: Front Public Health. 2024 Jul 11;12:1421053. doi: 10.3389/fpubh.2024.1421053 (PMC11269263; doi:10.3389/fpubh.2024.1421053)
Supplement: Supplementary file 3 [file Table_3.docx]

**Table S3.** Results of Tukey's post hoc between demographic factors and stress

| **Variables** | | | Mean Difference (I-J) | Std. Error | Sig. | | 95% Confidence Interval | | | |  |
| --- | --- | --- | --- | --- | --- | --- | --- | --- | --- | --- | --- |
|  |  |  |  |  |  |  | Lower Bound | | Upper Bound | |  |
| **Age group** | <30 | 30-50 | -.63537 | .91511 | | .767 | | -2.7882 | | 1.5174 |  |
|  |  | >50 | -2.12914 | .93390 | | .060 | | -4.3262 | | .0679 |  |
|  | 30-50 | <30 | .63537 | .91511 | | .767 | | -1.5174 | | 2.7882 |  |
|  |  | >50 | -1.49377^*^ | .41142 | | .001 | | -2.4617 | | -.5259 |  |
|  | >50 | <30 | 2.12914 | .93390 | | .060 | | -.0679 | | 4.3262 |  |
|  |  | 30-50 | 1.49377^*^ | .41142 | | .001 | | .5259 | | 2.4617 |  |
| **Education level** | Illiteracy | Elementary | -1.63702 | 1.29196 | | .803 | | -5.3375 | | 2.0634 |  |
|  |  | Middle school | -.63986 | 1.37417 | | .997 | | -4.5758 | | 3.2960 |  |
|  |  | High school | .84615 | 1.33433 | | .988 | | -2.9756 | | 4.6680 |  |
|  |  | Diploma | .04993 | 1.14992 | | 1.000 | | -3.2437 | | 3.3435 |  |
|  |  | Academic | .58242 | 1.12771 | | .996 | | -2.6476 | | 3.8124 |  |
|  | Elementary | Illiteracy | 1.63702 | 1.29196 | | .803 | | -2.0634 | | 5.3375 |  |
|  |  | Middle school | .99716 | 1.08793 | | .942 | | -2.1189 | | 4.1132 |  |
|  |  | High school | 2.48317 | 1.03715 | | .161 | | -.4874 | | 5.4538 |  |
|  |  | Diploma | 1.68695 | .78585 | | .266 | | -.5639 | | 3.9378 |  |
|  |  | Academic | 2.21944^*^ | .75298 | | .040 | | .0627 | | 4.3761 |  |
|  | Middle school | Illiteracy | .63986 | 1.37417 | | .997 | | -3.2960 | | 4.5758 |  |
|  |  | Elementary | -.99716 | 1.08793 | | .942 | | -4.1132 | | 2.1189 |  |
|  |  | High school | 1.48601 | 1.13792 | | .782 | | -1.7732 | | 4.7453 |  |
|  |  | Diploma | .68979 | .91473 | | .975 | | -1.9302 | | 3.3098 |  |
|  |  | Academic | 1.22228 | .88666 | | .740 | | -1.3173 | | 3.7619 |  |
|  | High school | Illiteracy | -.84615 | 1.33433 | | .988 | | -4.6680 | | 2.9756 |  |
|  |  | Elementary | -2.48317 | 1.03715 | | .161 | | -5.4538 | | .4874 |  |
|  |  | Middle school | -1.48601 | 1.13792 | | .782 | | -4.7453 | | 1.7732 |  |
|  |  | Diploma | -.79622 | .85372 | | .938 | | -3.2414 | | 1.6490 |  |
|  |  | Academic | -.26374 | .82357 | | 1.000 | | -2.6226 | | 2.0951 |  |
|  | Diploma | Illiteracy | -.04993 | 1.14992 | | 1.000 | | -3.3435 | | 3.2437 |  |
|  |  | Elementary | -1.68695 | .78585 | | .266 | | -3.9378 | | .5639 |  |
|  |  | Middle school | -.68979 | .91473 | | .975 | | -3.3098 | | 1.9302 |  |
|  |  | High school | .79622 | .85372 | | .938 | | -1.6490 | | 3.2414 |  |
|  |  | Academic | .53249 | .46919 | | .867 | | -.8114 | | 1.8763 |  |
|  | Academic | Illiteracy | -.58242 | 1.12771 | | .996 | | -3.8124 | | 2.6476 |  |
|  |  | Elementary | -2.21944^*^ | .75298 | | .040 | | -4.3761 | | -.0627 |  |
|  |  | Middle school | -1.22228 | .88666 | | .740 | | -3.7619 | | 1.3173 |  |
|  |  | High school | .26374 | .82357 | | 1.000 | | -2.0951 | | 2.6226 |  |
|  |  | Diploma | -.53249 | .46919 | | .867 | | -1.8763 | | .8114 |  |
| **Job** | Housewife | Employed | 1.18112 | .56930 | | .233 | | -.3792 | | 2.7415 |  |
|  |  | Retired | -.77388 | .66635 | | .773 | | -2.6002 | | 1.0525 |  |
|  |  | Self-employed | .86038 | .55928 | | .538 | | -.6725 | | 2.3933 |  |
|  |  | Labor | .10112 | .77945 | | 1.000 | | -2.0352 | | 2.2374 |  |
|  | Employed | Housewife | -1.18112 | .56930 | | .233 | | -2.7415 | | .3792 |  |
|  |  | Retired | -1.95500^*^ | .65204 | | .024 | | -3.7421 | | -.1679 |  |
|  |  | Self-employed | -.32074 | .54216 | | .976 | | -1.8067 | | 1.1652 |  |
|  |  | Labor | -1.08000 | .76725 | | .623 | | -3.1829 | | 1.0229 |  |
|  | Retired | Housewife | .77388 | .66635 | | .773 | | -1.0525 | | 2.6002 |  |
|  |  | Employed | 1.95500^*^ | .65204 | | .024 | | .1679 | | 3.7421 |  |
|  |  | Self-employed | 1.63426 | .64331 | | .084 | | -.1289 | | 3.3975 |  |
|  |  | Labor | .87500 | .84178 | | .837 | | -1.4322 | | 3.1822 |  |
|  | Self-employed | Housewife | -.86038 | .55928 | | .538 | | -2.3933 | | .6725 |  |
|  |  | Employed | .32074 | .54216 | | .976 | | -1.1652 | | 1.8067 |  |
|  |  | Retired | -1.63426 | .64331 | | .084 | | -3.3975 | | .1289 |  |
|  |  | Labor | -.75926 | .75985 | | .856 | | -2.8419 | | 1.3233 |  |
|  | labor | Housewife | -.10112 | .77945 | | 1.000 | | -2.2374 | | 2.0352 |  |
|  |  | Employed | 1.08000 | .76725 | | .623 | | -1.0229 | | 3.1829 |  |
|  |  | Retired | -.87500 | .84178 | | .837 | | -3.1822 | | 1.4322 |  |
|  |  | Self-employed | .75926 | .75985 | | .856 | | -1.3233 | | 2.8419 |  |
| **Duration of diabetes** | ≤ 5 | 6-10 | -.04452 | .49453 | | .996 | | -1.2084 | | 1.1194 |  |
|  |  | >10 | -1.58237^*^ | .50242 | | .005 | | -2.7648 | | -.3999 |  |
|  | 6-10 | ≤ 5 | .04452 | .49453 | | .996 | | -1.1194 | | 1.2084 |  |
|  |  | >10 | -1.53785^*^ | .55665 | | .017 | | -2.8479 | | -.2278 |  |
|  | >10 | ≤ 5 | 1.58237^*^ | .50242 | | .005 | | .3999 | | 2.7648 |  |
|  |  | 6-10 | 1.53785^*^ | .55665 | | .017 | | .2278 | | 2.8479 |  |
| **Method of obtaining health information** | Physician/ Health care providers | Internet | .43750 | .55281 | | .986 | | -1.2009 | | 2.0759 |  |
|  |  | Newspapers/magazines | -1.85662 | 1.04923 | | .570 | | -4.9663 | | 1.2531 |  |
|  |  | Friends and acquaintances | -.26061 | .69582 | | 1.000 | | -2.3229 | | 1.8017 |  |
|  |  | Book | -1.96250 | 1.10542 | | .566 | | -5.2387 | | 1.3137 |  |
|  |  | Radio, television and satellite | -1.12500 | .62119 | | .541 | | -2.9661 | | .7161 |  |
|  |  | I dont Know | .43750 | 1.10542 | | 1.000 | | -2.8387 | | 3.7137 |  |
|  | Internet | Physician/ Health care providers | -.43750 | .55281 | | .986 | | -2.0759 | | 1.2009 |  |
|  |  | Newspapers/magazines | -2.29412 | 1.01025 | | .261 | | -5.2883 | | .7001 |  |
|  |  | Friends and acquaintances | -.69811 | .63553 | | .928 | | -2.5817 | | 1.1855 |  |
|  |  | Book | -2.40000 | 1.06849 | | .273 | | -5.5668 | | .7668 |  |
|  |  | Radio, television and satellite | -1.56250 | .55281 | | .073 | | -3.2009 | | .0759 |  |
|  |  | I dont Know | .00000 | 1.06849 | | 1.000 | | -3.1668 | | 3.1668 |  |
|  | Newspapers/magazines | Physician/ Health care providers | 1.85662 | 1.04923 | | .570 | | -1.2531 | | 4.9663 |  |
|  |  | Internet | 2.29412 | 1.01025 | | .261 | | -.7001 | | 5.2883 |  |
|  |  | Friends and acquaintances | 1.59600 | 1.09507 | | .770 | | -1.6496 | | 4.8416 |  |
|  |  | Book | -.10588 | 1.39174 | | 1.000 | | -4.2307 | | 4.0190 |  |
|  |  | Radio, television and satellite | .73162 | 1.04923 | | .993 | | -2.3781 | | 3.8413 |  |
|  |  | I dont Know | 2.29412 | 1.39174 | | .651 | | -1.8307 | | 6.4190 |  |
|  | Friends and acquaintances | Physician/ Health care providers | .26061 | .69582 | | 1.000 | | -1.8017 | | 2.3229 |  |
|  |  | Internet | .69811 | .63553 | | .928 | | -1.1855 | | 2.5817 |  |
|  |  | Newspapers/magazines | -1.59600 | 1.09507 | | .770 | | -4.8416 | | 1.6496 |  |
|  |  | Book | -1.70189 | 1.14901 | | .756 | | -5.1073 | | 1.7036 |  |
|  |  | Radio, television and satellite | -.86439 | .69582 | | .877 | | -2.9267 | | 1.1979 |  |
|  |  | I dont Know | .69811 | 1.14901 | | .997 | | -2.7073 | | 4.1036 |  |
|  | Book | Physician/ Health care providers | 1.96250 | 1.10542 | | .566 | | -1.3137 | | 5.2387 |  |
|  |  | Internet | 2.40000 | 1.06849 | | .273 | | -.7668 | | 5.5668 |  |
|  |  | Newspapers/magazines | .10588 | 1.39174 | | 1.000 | | -4.0190 | | 4.2307 |  |
|  |  | Friends and acquaintances | 1.70189 | 1.14901 | | .756 | | -1.7036 | | 5.1073 |  |
|  |  | Radio, television and satellite | .83750 | 1.10542 | | .989 | | -2.4387 | | 4.1137 |  |
|  |  | I dont Know | 2.40000 | 1.43458 | | .635 | | -1.8518 | | 6.6518 |  |
|  | Radio, television and satellite | Physician/ Health care providers | 1.12500 | .62119 | | .541 | | -.7161 | | 2.9661 |  |
|  |  | Internet | 1.56250 | .55281 | | .073 | | -.0759 | | 3.2009 |  |
|  |  | Newspapers/magazines | -.73162 | 1.04923 | | .993 | | -3.8413 | | 2.3781 |  |
|  |  | Friends and acquaintances | .86439 | .69582 | | .877 | | -1.1979 | | 2.9267 |  |
|  |  | Book | -.83750 | 1.10542 | | .989 | | -4.1137 | | 2.4387 |  |
|  |  | I dont Know | 1.56250 | 1.10542 | | .794 | | -1.7137 | | 4.8387 |  |
|  | I do not know | Physician/ Health care providers | -.43750 | 1.10542 | | 1.000 | | -3.7137 | | 2.8387 |  |
|  |  | Internet | .00000 | 1.06849 | | 1.000 | | -3.1668 | | 3.1668 |  |
|  |  | Newspapers/magazines | -2.29412 | 1.39174 | | .651 | | -6.4190 | | 1.8307 |  |
|  |  | Friends and acquaintances | -.69811 | 1.14901 | | .997 | | -4.1036 | | 2.7073 |  |
|  |  | Book | -2.40000 | 1.43458 | | .635 | | -6.6518 | | 1.8518 |  |
|  |  | Radio, television and satellite | -1.56250 | 1.10542 | | .794 | | -4.8387 | | 1.7137 |  |
| **Method of obtaining information related to mental illness** | Physician/ Health care providers | Psychologist/Psychiatrist | -.57263 | 1.01624 | | .993 | | -3.4873 | | 2.3420 |  |
|  |  | Friends and acquaintances | -.09143 | .80997 | | 1.000 | | -2.4145 | | 2.2316 |  |
|  |  | Book | -1.52000 | 1.39581 | | .886 | | -5.5233 | | 2.4833 |  |
|  |  | Internet | -.10140 | .57455 | | 1.000 | | -1.7492 | | 1.5464 |  |
|  |  | Radio, television and satellite, TV | -2.25810^*^ | .76257 | | .038 | | -4.4452 | | -.0710 |  |
|  | Psychologist/Psychiatrist | Physician/ Health care providers | .57263 | 1.01624 | | .993 | | -2.3420 | | 3.4873 |  |
|  |  | Friends and acquaintances | .48120 | 1.12752 | | .998 | | -2.7526 | | 3.7150 |  |
|  |  | Book | -.94737 | 1.60111 | | .992 | | -5.5394 | | 3.6447 |  |
|  |  | Internet | .47124 | .97230 | | .997 | | -2.3174 | | 3.2598 |  |
|  |  | Radio, television and satellite, TV | -1.68546 | 1.09396 | | .638 | | -4.8230 | | 1.4521 |  |
|  | Friends and acquaintances | Physician/ Health care providers | .09143 | .80997 | | 1.000 | | -2.2316 | | 2.4145 |  |
|  |  | Psychologist/Psychiatrist | -.48120 | 1.12752 | | .998 | | -3.7150 | | 2.7526 |  |
|  |  | Book | -1.42857 | 1.47880 | | .928 | | -5.6699 | | 2.8127 |  |
|  |  | Internet | -.00997 | .75411 | | 1.000 | | -2.1728 | | 2.1528 |  |
|  |  | Radio, television and satellite, TV | -2.16667 | .90558 | | .162 | | -4.7639 | | .4306 |  |
|  | Book | Physician/ Health care providers | 1.52000 | 1.39581 | | .886 | | -2.4833 | | 5.5233 |  |
|  |  | Psychologist/Psychiatrist | .94737 | 1.60111 | | .992 | | -3.6447 | | 5.5394 |  |
|  |  | Friends and acquaintances | 1.42857 | 1.47880 | | .928 | | -2.8127 | | 5.6699 |  |
|  |  | Internet | 1.41860 | 1.36415 | | .904 | | -2.4939 | | 5.3311 |  |
|  |  | Radio, television and satellite, TV | -.73810 | 1.45338 | | .996 | | -4.9065 | | 3.4303 |  |
|  | Internet | Physician/ Health care providers | .10140 | .57455 | | 1.000 | | -1.5464 | | 1.7492 |  |
|  |  | Psychologist/Psychiatrist | -.47124 | .97230 | | .997 | | -3.2598 | | 2.3174 |  |
|  |  | Friends and acquaintances | .00997 | .75411 | | 1.000 | | -2.1528 | | 2.1728 |  |
|  |  | Book | -1.41860 | 1.36415 | | .904 | | -5.3311 | | 2.4939 |  |
|  |  | Radio, television and satellite, TV | -2.15670^*^ | .70294 | | .028 | | -4.1728 | | -.1406 |  |
|  | Radio, television and satellite | Physician/ Health care providers | 2.25810^*^ | .76257 | | .038 | | .0710 | | 4.4452 |  |
|  |  | Psychologist/Psychiatrist | 1.68546 | 1.09396 | | .638 | | -1.4521 | | 4.8230 |  |
|  |  | Friends and acquaintances | 2.16667 | .90558 | | .162 | | -.4306 | | 4.7639 |  |
|  |  | Book | .73810 | 1.45338 | | .996 | | -3.4303 | | 4.9065 |  |
|  |  | Internet | 2.15670^*^ | .70294 | | .028 | | .1406 | | 4.1728 |  |
| *. The mean difference is significant at the 0.05 level. | | | | | | | | | | | |
