# Supplementary material for: Depression literacy, mental health literacy, and their relationship with psychological status and quality of life in patients with type 2 diabetes mellitus
Source: Front Public Health. 2024 Jul 11;12:1421053. doi: 10.3389/fpubh.2024.1421053 (PMC11269263; doi:10.3389/fpubh.2024.1421053)
Supplement: Supplementary file 4 [file Table_4.docx]

**Table S4.** Results of Tukey's post hoc between demographic factors and mental health literacy (MHL

| Variables | | | Mean Difference (I-J) | Std. Error | Sig. | 95% Confidence Interval | | |  |
| --- | --- | --- | --- | --- | --- | --- | --- | --- | --- |
|  |  |  |  |  |  | Lower Bound | Upper Bound |  |  |
| **Age group** | <30 | 30-50 | 1.50847 | 2.09523 | .752 | -3.4206 | 6.4376 |  |  |
|  |  | >50 | 5.31636^*^ | 2.13825 | .035 | .2860 | 10.3467 |  |  |
|  | 30-50 | <30 | -1.50847 | 2.09523 | .752 | -6.4376 | 3.4206 |  |  |
|  |  | >50 | 3.80789^*^ | .94200 | .000 | 1.5918 | 6.0240 |  |  |
|  | >50 | <30 | -5.31636^*^ | 2.13825 | .035 | -10.3467 | -.2860 |  |  |
|  |  | 30-50 | -3.80789^*^ | .94200 | .000 | -6.0240 | -1.5918 |  |  |
| **Education level** | Illiteracy | Elementary | .67642 | 3.00235 | 1.000 | -7.9229 | 9.2758 |  |  |
|  |  | Middle school | -2.04306 | 3.19340 | .988 | -11.1896 | 7.1035 |  |  |
|  |  | High school | -3.60808 | 3.10081 | .854 | -12.4895 | 5.2733 |  |  |
|  |  | Diploma | -2.16332 | 2.67226 | .966 | -9.8172 | 5.4906 |  |  |
|  |  | Academic | -2.60473 | 2.62067 | .920 | -10.1109 | 4.9014 |  |  |
|  | Elementary | Illiteracy | -.67642 | 3.00235 | 1.000 | -9.2758 | 7.9229 |  |  |
|  |  | Middle school | -2.71948 | 2.52820 | .891 | -9.9608 | 4.5218 |  |  |
|  |  | High school | -4.28450 | 2.41021 | .481 | -11.1878 | 2.6188 |  |  |
|  |  | Diploma | -2.83974 | 1.82621 | .629 | -8.0704 | 2.3909 |  |  |
|  |  | Academic | -3.28115 | 1.74984 | .419 | -8.2931 | 1.7307 |  |  |
|  | Middle school | Illiteracy | 2.04306 | 3.19340 | .988 | -7.1035 | 11.1896 |  |  |
|  |  | Elementary | 2.71948 | 2.52820 | .891 | -4.5218 | 9.9608 |  |  |
|  |  | High school | -1.56502 | 2.64438 | .992 | -9.1391 | 6.0090 |  |  |
|  |  | Diploma | -.12025 | 2.12573 | 1.000 | -6.2088 | 5.9683 |  |  |
|  |  | Academic | -.56167 | 2.06049 | 1.000 | -6.4633 | 5.3400 |  |  |
|  | High school | Illiteracy | 3.60808 | 3.10081 | .854 | -5.2733 | 12.4895 |  |  |
|  |  | Elementary | 4.28450 | 2.41021 | .481 | -2.6188 | 11.1878 |  |  |
|  |  | Middle school | 1.56502 | 2.64438 | .992 | -6.0090 | 9.1391 |  |  |
|  |  | Diploma | 1.44476 | 1.98393 | .978 | -4.2376 | 7.1272 |  |  |
|  |  | Academic | 1.00335 | 1.91386 | .995 | -4.4784 | 6.4851 |  |  |
|  | Diploma | Illiteracy | 2.16332 | 2.67226 | .966 | -5.4906 | 9.8172 |  |  |
|  |  | Elementary | 2.83974 | 1.82621 | .629 | -2.3909 | 8.0704 |  |  |
|  |  | Middle school | .12025 | 2.12573 | 1.000 | -5.9683 | 6.2088 |  |  |
|  |  | High school | -1.44476 | 1.98393 | .978 | -7.1272 | 4.2376 |  |  |
|  |  | Academic | -.44142 | 1.09033 | .999 | -3.5644 | 2.6815 |  |  |
|  | Academic | Illiteracy | 2.60473 | 2.62067 | .920 | -4.9014 | 10.1109 |  |  |
|  |  | Elementary | 3.28115 | 1.74984 | .419 | -1.7307 | 8.2931 |  |  |
|  |  | Middle school | .56167 | 2.06049 | 1.000 | -5.3400 | 6.4633 |  |  |
|  |  | High school | -1.00335 | 1.91386 | .995 | -6.4851 | 4.4784 |  |  |
|  |  | Diploma | .44142 | 1.09033 | .999 | -2.6815 | 3.5644 |  |  |
| **Job** | Housewife | Employed | -1.06520 | 1.29888 | .924 | -4.6252 | 2.4948 |  |  |
|  |  | Retired | 2.48650 | 1.52029 | .476 | -1.6804 | 6.6534 |  |  |
|  |  | Self-employed | -2.29109 | 1.27602 | .378 | -5.7884 | 1.2063 |  |  |
|  |  | Labor | 1.71115 | 1.77834 | .872 | -3.1630 | 6.5853 |  |  |
|  | Employed | Housewife | 1.06520 | 1.29888 | .924 | -2.4948 | 4.6252 |  |  |
|  |  | Retired | 3.55170 | 1.48765 | .121 | -.5257 | 7.6291 |  |  |
|  |  | Self-employed | -1.22588 | 1.23695 | .859 | -4.6161 | 2.1644 |  |  |
|  |  | Labor | 2.77636 | 1.75051 | .507 | -2.0215 | 7.5742 |  |  |
|  | Retired | Housewife | -2.48650 | 1.52029 | .476 | -6.6534 | 1.6804 |  |  |
|  |  | Employed | -3.55170 | 1.48765 | .121 | -7.6291 | .5257 |  |  |
|  |  | Self-employed | -4.77759^*^ | 1.46774 | .011 | -8.8004 | -.7548 |  |  |
|  |  | Labor | -.77535 | 1.92055 | .994 | -6.0392 | 4.4885 |  |  |
|  | Self-employed | Housewife | 2.29109 | 1.27602 | .378 | -1.2063 | 5.7884 |  |  |
|  |  | Employed | 1.22588 | 1.23695 | .859 | -2.1644 | 4.6161 |  |  |
|  |  | Retired | 4.77759^*^ | 1.46774 | .011 | .7548 | 8.8004 |  |  |
|  |  | Labor | 4.00224 | 1.73362 | .144 | -.7493 | 8.7538 |  |  |
|  | labor | Housewife | -1.71115 | 1.77834 | .872 | -6.5853 | 3.1630 |  |  |
|  |  | Employed | -2.77636 | 1.75051 | .507 | -7.5742 | 2.0215 |  |  |
|  |  | Retired | .77535 | 1.92055 | .994 | -4.4885 | 6.0392 |  |  |
|  |  | Self-employed | -4.00224 | 1.73362 | .144 | -8.7538 | .7493 |  |  |
| **Duration of diabetes** | ≤ 5 | 6-10 | 1.71273 | 1.14974 | .297 | -.9932 | 4.4187 |  |  |
|  |  | >10 | 3.10954^*^ | 1.16808 | .022 | .3604 | 5.8587 |  |  |
|  | 6-10 | ≤ 5 | -1.71273 | 1.14974 | .297 | -4.4187 | .9932 |  |  |
|  |  | >10 | 1.39681 | 1.29416 | .528 | -1.6490 | 4.4427 |  |  |
|  | >10 | ≤ 5 | -3.10954^*^ | 1.16808 | .022 | -5.8587 | -.3604 |  |  |
|  |  | 6-10 | -1.39681 | 1.29416 | .528 | -4.4427 | 1.6490 |  |  |
| **Method of obtaining health information** | Physician/ Health care providers | Internet | -.55531 | 1.25722 | .999 | -4.2815 | 3.1708 |  |  |
|  |  | Newspapers/magazines | 3.36559 | 2.38618 | .796 | -3.7066 | 10.4378 |  |  |
|  |  | Friends and acquaintances | 2.84692 | 1.58245 | .549 | -1.8431 | 7.5370 |  |  |
|  |  | Book | 4.20180 | 2.51395 | .636 | -3.2491 | 11.6527 |  |  |
|  |  | Radio, television and satellite | 3.96391 | 1.41272 | .077 | -.2231 | 8.1509 |  |  |
|  |  | I dont Know | 8.59573^*^ | 2.51395 | .012 | 1.1449 | 16.0466 |  |  |
|  | Internet | Physician/ Health care providers | .55531 | 1.25722 | .999 | -3.1708 | 4.2815 |  |  |
|  |  | Newspapers/magazines | 3.92091 | 2.29753 | .612 | -2.8885 | 10.7303 |  |  |
|  |  | Friends and acquaintances | 3.40223 | 1.44532 | .221 | -.8814 | 7.6859 |  |  |
|  |  | Book | 4.75712 | 2.42998 | .444 | -2.4449 | 11.9591 |  |  |
|  |  | Radio, television and satellite | 4.51922^*^ | 1.25722 | .007 | .7931 | 8.2454 |  |  |
|  |  | I dont Know | 9.15104^*^ | 2.42998 | .004 | 1.9491 | 16.3530 |  |  |
|  | Newspapers/ magazines | Physician/ Health care providers | -3.36559 | 2.38618 | .796 | -10.4378 | 3.7066 |  |  |
|  |  | Internet | -3.92091 | 2.29753 | .612 | -10.7303 | 2.8885 |  |  |
|  |  | Friends and acquaintances | -.51868 | 2.49042 | 1.000 | -7.8998 | 6.8624 |  |  |
|  |  | Book | .83621 | 3.16513 | 1.000 | -8.5446 | 10.2170 |  |  |
|  |  | Radio, television and satellite | .59831 | 2.38618 | 1.000 | -6.4739 | 7.6705 |  |  |
|  |  | I dont Know | 5.23013 | 3.16513 | .648 | -4.1507 | 14.6110 |  |  |
|  | Friends and acquaintances | Physician/ Health care providers | -2.84692 | 1.58245 | .549 | -7.5370 | 1.8431 |  |  |
|  |  | Internet | -3.40223 | 1.44532 | .221 | -7.6859 | .8814 |  |  |
|  |  | Newspapers/magazines | .51868 | 2.49042 | 1.000 | -6.8624 | 7.8998 |  |  |
|  |  | Book | 1.35489 | 2.61311 | .999 | -6.3899 | 9.0996 |  |  |
|  |  | Radio, television and satellite | 1.11699 | 1.58245 | .992 | -3.5731 | 5.8071 |  |  |
|  |  | I dont Know | 5.74881 | 2.61311 | .298 | -1.9959 | 13.4935 |  |  |
|  | Book | Physician/ Health care providers | -4.20180 | 2.51395 | .636 | -11.6527 | 3.2491 |  |  |
|  |  | Internet | -4.75712 | 2.42998 | .444 | -11.9591 | 2.4449 |  |  |
|  |  | Newspapers/magazines | -.83621 | 3.16513 | 1.000 | -10.2170 | 8.5446 |  |  |
|  |  | Friends and acquaintances | -1.35489 | 2.61311 | .999 | -9.0996 | 6.3899 |  |  |
|  |  | Radio, television and satellite | -.23790 | 2.51395 | 1.000 | -7.6888 | 7.2130 |  |  |
|  |  | I dont Know | 4.39392 | 3.26254 | .829 | -5.2756 | 14.0635 |  |  |
|  | Radio, television and satellite | Physician/ Health care providers | -3.96391 | 1.41272 | .077 | -8.1509 | .2231 |  |  |
|  |  | Internet | -4.51922^*^ | 1.25722 | .007 | -8.2454 | -.7931 |  |  |
|  |  | Newspapers/magazines | -.59831 | 2.38618 | 1.000 | -7.6705 | 6.4739 |  |  |
|  |  | Friends and acquaintances | -1.11699 | 1.58245 | .992 | -5.8071 | 3.5731 |  |  |
|  |  | Book | .23790 | 2.51395 | 1.000 | -7.2130 | 7.6888 |  |  |
|  |  | I dont Know | 4.63182 | 2.51395 | .520 | -2.8190 | 12.0827 |  |  |
|  | I do not know | Physician/ Health care providers | -8.59573^*^ | 2.51395 | .012 | -16.0466 | -1.1449 |  |  |
|  |  | Internet | -9.15104^*^ | 2.42998 | .004 | -16.3530 | -1.9491 |  |  |
|  |  | Newspapers/magazines | -5.23013 | 3.16513 | .648 | -14.6110 | 4.1507 |  |  |
|  |  | Friends and acquaintances | -5.74881 | 2.61311 | .298 | -13.4935 | 1.9959 |  |  |
|  |  | Book | -4.39392 | 3.26254 | .829 | -14.0635 | 5.2756 |  |  |
|  |  | Radio, television and satellite | -4.63182 | 2.51395 | .520 | -12.0827 | 2.8190 |  |  |
| **Method of obtaining information related to mental illness** | Physician/ Health care providers | Psychologist/Psychiatrist | -.23911 | 2.33796 | 1.000 | -6.9445 | 6.4663 |  |  |
|  |  | Friends and acquaintances | 3.70485 | 1.86342 | .351 | -1.6395 | 9.0492 |  |  |
|  |  | Book | .97832 | 3.21120 | 1.000 | -8.2316 | 10.1882 |  |  |
|  |  | Internet | 2.00618 | 1.32181 | .653 | -1.7848 | 5.7972 |  |  |
|  |  | Radio, television and satellite, TV | 6.07378^*^ | 1.75435 | .008 | 1.0422 | 11.1054 |  |  |
|  | Psychologist/Psychiatrist | Physician/ Health care providers | .23911 | 2.33796 | 1.000 | -6.4663 | 6.9445 |  |  |
|  |  | Friends and acquaintances | 3.94396 | 2.59398 | .651 | -3.4957 | 11.3836 |  |  |
|  |  | Book | 1.21744 | 3.68350 | .999 | -9.3470 | 11.7819 |  |  |
|  |  | Internet | 2.24529 | 2.23686 | .916 | -4.1701 | 8.6607 |  |  |
|  |  | Radio, television and satellite, TV | 6.31290 | 2.51677 | .125 | -.9053 | 13.5311 |  |  |
|  | Friends and acquaintances | Physician/ Health care providers | -3.70485 | 1.86342 | .351 | -9.0492 | 1.6395 |  |  |
|  |  | Psychologist/Psychiatrist | -3.94396 | 2.59398 | .651 | -11.3836 | 3.4957 |  |  |
|  |  | Book | -2.72652 | 3.40213 | .967 | -12.4840 | 7.0310 |  |  |
|  |  | Internet | -1.69867 | 1.73489 | .924 | -6.6744 | 3.2771 |  |  |
|  |  | Radio, television and satellite, TV | 2.36894 | 2.08337 | .866 | -3.6063 | 8.3442 |  |  |
|  | Book | Physician/ Health care providers | -.97832 | 3.21120 | 1.000 | -10.1882 | 8.2316 |  |  |
|  |  | Psychologist/Psychiatrist | -1.21744 | 3.68350 | .999 | -11.7819 | 9.3470 |  |  |
|  |  | Friends and acquaintances | 2.72652 | 3.40213 | .967 | -7.0310 | 12.4840 |  |  |
|  |  | Internet | 1.02785 | 3.13836 | .999 | -7.9731 | 10.0288 |  |  |
|  |  | Radio, television and satellite, TV | 5.09546 | 3.34364 | .649 | -4.4943 | 14.6852 |  |  |
|  | Internet | Physician/ Health care providers | -2.00618 | 1.32181 | .653 | -5.7972 | 1.7848 |  |  |
|  |  | Psychologist/Psychiatrist | -2.24529 | 2.23686 | .916 | -8.6607 | 4.1701 |  |  |
|  |  | Friends and acquaintances | 1.69867 | 1.73489 | .924 | -3.2771 | 6.6744 |  |  |
|  |  | Book | -1.02785 | 3.13836 | .999 | -10.0288 | 7.9731 |  |  |
|  |  | Radio, television and satellite, TV | 4.06761 | 1.61718 | .123 | -.5705 | 8.7058 |  |  |
|  | Radio, television and satellite | Physician/ Health care providers | -6.07378^*^ | 1.75435 | .008 | -11.1054 | -1.0422 |  |  |
|  |  | Psychologist/Psychiatrist | -6.31290 | 2.51677 | .125 | -13.5311 | .9053 |  |  |
|  |  | Friends and acquaintances | -2.36894 | 2.08337 | .866 | -8.3442 | 3.6063 |  |  |
|  |  | Book | -5.09546 | 3.34364 | .649 | -14.6852 | 4.4943 |  |  |
|  |  | Internet | -4.06761 | 1.61718 | .123 | -8.7058 | .5705 |  |  |
| *. The mean difference is significant at the 0.05 level. | | | | | | | | | |
