# Supplementary material for: Depression literacy, mental health literacy, and their relationship with psychological status and quality of life in patients with type 2 diabetes mellitus
Source: Front Public Health. 2024 Jul 11;12:1421053. doi: 10.3389/fpubh.2024.1421053 (PMC11269263; doi:10.3389/fpubh.2024.1421053)
Supplement: Supplementary file 5 [file Table_5.docx]

**Table S5.** Results of Tukey's post hoc between demographic factors and depression literacy (D-Lit)

| **Variables** | | | Mean Difference (I-J) | Std. Error | Sig. | 95% Confidence Interval | |  |
| --- | --- | --- | --- | --- | --- | --- | --- | --- |
|  |  |  |  |  |  | Lower Bound | Upper Bound |  |
| **Age group** | <30 | 30-50 | 1.26463 | .98672 | .406 | -1.0567 | 3.5859 |  |
|  |  | >50 | 2.59868^*^ | 1.00699 | .028 | .2297 | 4.9676 |  |
|  | 30-50 | <30 | -1.26463 | .98672 | .406 | -3.5859 | 1.0567 |  |
|  |  | >50 | 1.33405^*^ | .44362 | .008 | .2904 | 2.3777 |  |
|  | >50 | <30 | -2.59868^*^ | 1.00699 | .028 | -4.9676 | -.2297 |  |
|  |  | 30-50 | -1.33405^*^ | .44362 | .008 | -2.3777 | -.2904 |  |
| **Education level** | Illiteracy | Elementary | .65625 | 1.37638 | .997 | -3.2860 | 4.5985 |  |
|  |  | Middle school | -1.00000 | 1.46396 | .984 | -5.1931 | 3.1931 |  |
|  |  | High school | -1.92308 | 1.42152 | .755 | -5.9946 | 2.1484 |  |
|  |  | Diploma | -1.34211 | 1.22506 | .883 | -4.8509 | 2.1667 |  |
|  |  | Academic | -2.56593 | 1.20140 | .271 | -6.0070 | .8751 |  |
|  | Elementary | Illiteracy | -.65625 | 1.37638 | .997 | -4.5985 | 3.2860 |  |
|  |  | Middle school | -1.65625 | 1.15902 | .709 | -4.9759 | 1.6634 |  |
|  |  | High school | -2.57933 | 1.10492 | .183 | -5.7440 | .5854 |  |
|  |  | Diploma | -1.99836 | .83720 | .163 | -4.3963 | .3996 |  |
|  |  | Academic | -3.22218^*^ | .80219 | .001 | -5.5198 | -.9246 |  |
|  | Middle school | Illiteracy | 1.00000 | 1.46396 | .984 | -3.1931 | 5.1931 |  |
|  |  | Elementary | 1.65625 | 1.15902 | .709 | -1.6634 | 4.9759 |  |
|  |  | High school | -.92308 | 1.21228 | .974 | -4.3953 | 2.5491 |  |
|  |  | Diploma | -.34211 | .97451 | .999 | -3.1333 | 2.4491 |  |
|  |  | Academic | -1.56593 | .94460 | .561 | -4.2715 | 1.1396 |  |
|  | High school | Illiteracy | 1.92308 | 1.42152 | .755 | -2.1484 | 5.9946 |  |
|  |  | Elementary | 2.57933 | 1.10492 | .183 | -.5854 | 5.7440 |  |
|  |  | Middle school | .92308 | 1.21228 | .974 | -2.5491 | 4.3953 |  |
|  |  | Diploma | .58097 | .90950 | .988 | -2.0240 | 3.1860 |  |
|  |  | Academic | -.64286 | .87738 | .978 | -3.1559 | 1.8701 |  |
|  | Diploma | Illiteracy | 1.34211 | 1.22506 | .883 | -2.1667 | 4.8509 |  |
|  |  | Elementary | 1.99836 | .83720 | .163 | -.3996 | 4.3963 |  |
|  |  | Middle school | .34211 | .97451 | .999 | -2.4491 | 3.1333 |  |
|  |  | High school | -.58097 | .90950 | .988 | -3.1860 | 2.0240 |  |
|  |  | Academic | -1.22383 | .49985 | .143 | -2.6555 | .2078 |  |
|  | Academic | Illiteracy | 2.56593 | 1.20140 | .271 | -.8751 | 6.0070 |  |
|  |  | Elementary | 3.22218^*^ | .80219 | .001 | .9246 | 5.5198 |  |
|  |  | Middle school | 1.56593 | .94460 | .561 | -1.1396 | 4.2715 |  |
|  |  | High school | .64286 | .87738 | .978 | -1.8701 | 3.1559 |  |
|  |  | Diploma | 1.22383 | .49985 | .143 | -.2078 | 2.6555 |  |
| **Job** | Housewife | Employed | -.98022 | .62367 | .517 | -2.6896 | .7292 |  |
|  |  | Retired | .62620 | .72999 | .912 | -1.3746 | 2.6270 |  |
|  |  | Self-employed | -1.30171 | .61270 | .212 | -2.9810 | .3776 |  |
|  |  | Labor | .12263 | .85389 | 1.000 | -2.2177 | 2.4630 |  |
|  | Employed | Housewife | .98022 | .62367 | .517 | -.7292 | 2.6896 |  |
|  |  | Retired | 1.60643 | .71432 | .164 | -.3514 | 3.5642 |  |
|  |  | Self-employed | -.32148 | .59394 | .983 | -1.9494 | 1.3064 |  |
|  |  | Labor | 1.10286 | .84053 | .684 | -1.2009 | 3.4066 |  |
|  | Retired | Housewife | -.62620 | .72999 | .912 | -2.6270 | 1.3746 |  |
|  |  | Employed | -1.60643 | .71432 | .164 | -3.5642 | .3514 |  |
|  |  | Self-employed | -1.92791 | .70476 | .051 | -3.8595 | .0037 |  |
|  |  | Labor | -.50357 | .92218 | .982 | -3.0311 | 2.0240 |  |
|  | Self-employed | Housewife | 1.30171 | .61270 | .212 | -.3776 | 2.9810 |  |
|  |  | Employed | .32148 | .59394 | .983 | -1.3064 | 1.9494 |  |
|  |  | Retired | 1.92791 | .70476 | .051 | -.0037 | 3.8595 |  |
|  |  | Labor | 1.42434 | .83242 | .428 | -.8572 | 3.7059 |  |
|  | labor | Housewife | -.12263 | .85389 | 1.000 | -2.4630 | 2.2177 |  |
|  |  | Employed | -1.10286 | .84053 | .684 | -3.4066 | 1.2009 |  |
|  |  | Retired | .50357 | .92218 | .982 | -2.0240 | 3.0311 |  |
|  |  | Self-employed | -1.42434 | .83242 | .428 | -3.7059 | .8572 |  |
| **Duration of diabetes** | ≤ 5 | 6-10 | .38280 | .53205 | .752 | -.8694 | 1.6350 |  |
|  |  | >10 | 1.26956 | .54054 | .051 | -.0026 | 2.5417 |  |
|  | 6-10 | ≤ 5 | -.38280 | .53205 | .752 | -1.6350 | .8694 |  |
|  |  | >10 | .88676 | .59888 | .301 | -.5227 | 2.2962 |  |
|  | >10 | ≤ 5 | -1.26956 | .54054 | .051 | -2.5417 | .0026 |  |
|  |  | 6-10 | -.88676 | .59888 | .301 | -2.2962 | .5227 |  |
| **Method of obtaining health information** | Physician/ Health care providers | Internet | -1.77153^*^ | .58890 | .044 | -3.5169 | -.0262 |  |
|  |  | Newspapers/magazines | -.57059 | 1.11771 | .999 | -3.8833 | 2.7421 |  |
|  |  | Friends and acquaintances | .44717 | .74124 | .997 | -1.7497 | 2.6441 |  |
|  |  | Book | -2.56667 | 1.17757 | .309 | -6.0567 | .9234 |  |
|  |  | Radio, television and satellite | -1.06250 | .66174 | .679 | -3.0238 | .8988 |  |
|  |  | I dont Know | 2.36667 | 1.17757 | .410 | -1.1234 | 5.8567 |  |
|  | Internet | Physician/ Health care providers | 1.77153^*^ | .58890 | .044 | .0262 | 3.5169 |  |
|  |  | Newspapers/magazines | 1.20094 | 1.07619 | .923 | -1.9887 | 4.3906 |  |
|  |  | Friends and acquaintances | 2.21870^*^ | .67701 | .019 | .2122 | 4.2252 |  |
|  |  | Book | -.79513 | 1.13823 | .993 | -4.1686 | 2.5784 |  |
|  |  | Radio, television and satellite | .70903 | .58890 | .892 | -1.0363 | 2.4544 |  |
|  |  | I dont Know | 4.13820^*^ | 1.13823 | .006 | .7647 | 7.5117 |  |
|  | Newspapers/ magazines | Physician/ Health care providers | .57059 | 1.11771 | .999 | -2.7421 | 3.8833 |  |
|  |  | Internet | -1.20094 | 1.07619 | .923 | -4.3906 | 1.9887 |  |
|  |  | Friends and acquaintances | 1.01776 | 1.16654 | .976 | -2.4396 | 4.4752 |  |
|  |  | Book | -1.99608 | 1.48258 | .830 | -6.3902 | 2.3980 |  |
|  |  | Radio, television and satellite | -.49191 | 1.11771 | .999 | -3.8046 | 2.8208 |  |
|  |  | I dont Know | 2.93725 | 1.48258 | .428 | -1.4568 | 7.3313 |  |
|  | Friends and acquaintances | Physician/ Health care providers | -.44717 | .74124 | .997 | -2.6441 | 1.7497 |  |
|  |  | Internet | -2.21870^*^ | .67701 | .019 | -4.2252 | -.2122 |  |
|  |  | Newspapers/magazines | -1.01776 | 1.16654 | .976 | -4.4752 | 2.4396 |  |
|  |  | Book | -3.01384 | 1.22401 | .176 | -6.6416 | .6139 |  |
|  |  | Radio, television and satellite | -1.50967 | .74124 | .393 | -3.7066 | .6872 |  |
|  |  | I dont Know | 1.91950 | 1.22401 | .703 | -1.7082 | 5.5472 |  |
|  | Book | Physician/ Health care providers | 2.56667 | 1.17757 | .309 | -.9234 | 6.0567 |  |
|  |  | Internet | .79513 | 1.13823 | .993 | -2.5784 | 4.1686 |  |
|  |  | Newspapers/magazines | 1.99608 | 1.48258 | .830 | -2.3980 | 6.3902 |  |
|  |  | Friends and acquaintances | 3.01384 | 1.22401 | .176 | -.6139 | 6.6416 |  |
|  |  | Radio, television and satellite | 1.50417 | 1.17757 | .862 | -1.9859 | 4.9942 |  |
|  |  | I dont Know | 4.93333^*^ | 1.52821 | .023 | .4040 | 9.4627 |  |
|  | Radio, television and satellite | Physician/ Health care providers | 1.06250 | .66174 | .679 | -.8988 | 3.0238 |  |
|  |  | Internet | -.70903 | .58890 | .892 | -2.4544 | 1.0363 |  |
|  |  | Newspapers/magazines | .49191 | 1.11771 | .999 | -2.8208 | 3.8046 |  |
|  |  | Friends and acquaintances | 1.50967 | .74124 | .393 | -.6872 | 3.7066 |  |
|  |  | Book | -1.50417 | 1.17757 | .862 | -4.9942 | 1.9859 |  |
|  |  | I dont Know | 3.42917 | 1.17757 | .058 | -.0609 | 6.9192 |  |
|  | I do not know | Physician/ Health care providers | -2.36667 | 1.17757 | .410 | -5.8567 | 1.1234 |  |
|  |  | Internet | -4.13820^*^ | 1.13823 | .006 | -7.5117 | -.7647 |  |
|  |  | Newspapers/magazines | -2.93725 | 1.48258 | .428 | -7.3313 | 1.4568 |  |
|  |  | Friends and acquaintances | -1.91950 | 1.22401 | .703 | -5.5472 | 1.7082 |  |
|  |  | Book | -4.93333^*^ | 1.52821 | .023 | -9.4627 | -.4040 |  |
|  |  | Radio, television and satellite | -3.42917 | 1.17757 | .058 | -6.9192 | .0609 |  |
| **Method of obtaining information related to mental illness** | Physician/ Health care providers | Psychologist/Psychiatrist | -.22246 | 1.07753 | 1.000 | -3.3129 | 2.8680 |  |
|  |  | Friends and acquaintances | -.42095 | .85883 | .996 | -2.8841 | 2.0422 |  |
|  |  | Book | -2.57333 | 1.48000 | .507 | -6.8181 | 1.6714 |  |
|  |  | Internet | -2.04620^*^ | .60921 | .011 | -3.7934 | -.2990 |  |
|  |  | Radio, television and satellite, TV | .37905 | .80856 | .997 | -1.9399 | 2.6980 |  |
|  | Psychologist/Psychiatrist | Physician/ Health care providers | .22246 | 1.07753 | 1.000 | -2.8680 | 3.3129 |  |
|  |  | Friends and acquaintances | -.19850 | 1.19553 | 1.000 | -3.6273 | 3.2303 |  |
|  |  | Book | -2.35088 | 1.69768 | .736 | -7.2199 | 2.5182 |  |
|  |  | Internet | -1.82375 | 1.03094 | .487 | -4.7805 | 1.1330 |  |
|  |  | Radio, television and satellite, TV | .60150 | 1.15995 | .995 | -2.7253 | 3.9283 |  |
|  | Friends and acquaintances | Physician/ Health care providers | .42095 | .85883 | .996 | -2.0422 | 2.8841 |  |
|  |  | Psychologist/Psychiatrist | .19850 | 1.19553 | 1.000 | -3.2303 | 3.6273 |  |
|  |  | Book | -2.15238 | 1.56800 | .743 | -6.6495 | 2.3447 |  |
|  |  | Internet | -1.62525 | .79959 | .326 | -3.9185 | .6680 |  |
|  |  | Radio, television and satellite, TV | .80000 | .96020 | .961 | -1.9539 | 3.5539 |  |
|  | Book | Physician/ Health care providers | 2.57333 | 1.48000 | .507 | -1.6714 | 6.8181 |  |
|  |  | Psychologist/Psychiatrist | 2.35088 | 1.69768 | .736 | -2.5182 | 7.2199 |  |
|  |  | Friends and acquaintances | 2.15238 | 1.56800 | .743 | -2.3447 | 6.6495 |  |
|  |  | Internet | .52713 | 1.44643 | .999 | -3.6213 | 4.6756 |  |
|  |  | Radio, television and satellite, TV | 2.95238 | 1.54104 | .394 | -1.4674 | 7.3722 |  |
|  | Internet | Physician/ Health care providers | 2.04620^*^ | .60921 | .011 | .2990 | 3.7934 |  |
|  |  | Psychologist/Psychiatrist | 1.82375 | 1.03094 | .487 | -1.1330 | 4.7805 |  |
|  |  | Friends and acquaintances | 1.62525 | .79959 | .326 | -.6680 | 3.9185 |  |
|  |  | Book | -.52713 | 1.44643 | .999 | -4.6756 | 3.6213 |  |
|  |  | Radio, television and satellite, TV | 2.42525^*^ | .74534 | .016 | .2876 | 4.5629 |  |
|  | Radio, television and satellite | Physician/ Health care providers | -.37905 | .80856 | .997 | -2.6980 | 1.9399 |  |
|  |  | Psychologist/Psychiatrist | -.60150 | 1.15995 | .995 | -3.9283 | 2.7253 |  |
|  |  | Friends and acquaintances | -.80000 | .96020 | .961 | -3.5539 | 1.9539 |  |
|  |  | Book | -2.95238 | 1.54104 | .394 | -7.3722 | 1.4674 |  |
|  |  | Internet | -2.42525^*^ | .74534 | .016 | -4.5629 | -.2876 |  |
|  | *. The mean difference is significant at the 0.05 level. | | | | | | | |
