# Supplementary material for: Depression literacy, mental health literacy, and their relationship with psychological status and quality of life in patients with type 2 diabetes mellitus
Source: Front Public Health. 2024 Jul 11;12:1421053. doi: 10.3389/fpubh.2024.1421053 (PMC11269263; doi:10.3389/fpubh.2024.1421053)
Supplement: Supplementary file 6 [file Table_6.docx]

**Table S6.** Results of Tukey's post hoc between demographic factors and Diabetes quality of life (DQOL)

| Variables | | | Mean Difference (I-J) | Std. Error | Sig. | 95% Confidence Interval | |  |
| --- | --- | --- | --- | --- | --- | --- | --- | --- |
|  |  |  |  |  |  | Lower Bound | Upper Bound |  |
| **Age group** | <30 | 30-50 | .91826 | 1.71512 | .854 | -3.1166 | 4.9531 |  |
|  |  | >50 | 1.09304 | 1.75034 | .807 | -3.0247 | 5.2108 |  |
|  | 30-50 | <30 | -.91826 | 1.71512 | .854 | -4.9531 | 3.1166 |  |
|  |  | >50 | .17477 | .77110 | .972 | -1.6393 | 1.9888 |  |
|  | >50 | <30 | -1.09304 | 1.75034 | .807 | -5.2108 | 3.0247 |  |
|  |  | 30-50 | -.17477 | .77110 | .972 | -1.9888 | 1.6393 |  |
| **Education level** | Illiteracy | Elementary | 2.18503 | 2.37642 | .941 | -4.6215 | 8.9916 |  |
|  |  | Middle school | 2.24913 | 2.52764 | .949 | -4.9906 | 9.4888 |  |
|  |  | High school | 1.73077 | 2.45436 | .981 | -5.2990 | 8.7606 |  |
|  |  | Diploma | 3.60631 | 2.11515 | .529 | -2.4519 | 9.6646 |  |
|  |  | Academic | 1.18545 | 2.07431 | .993 | -4.7558 | 7.1267 |  |
|  | Elementary | Illiteracy | -2.18503 | 2.37642 | .941 | -8.9916 | 4.6215 |  |
|  |  | Middle school | .06409 | 2.00113 | 1.000 | -5.6676 | 5.7957 |  |
|  |  | High school | -.45427 | 1.90773 | 1.000 | -5.9184 | 5.0099 |  |
|  |  | Diploma | 1.42127 | 1.44548 | .923 | -2.7189 | 5.5614 |  |
|  |  | Academic | -.99959 | 1.38503 | .979 | -4.9666 | 2.9674 |  |
|  | Middle school | Illiteracy | -2.24913 | 2.52764 | .949 | -9.4888 | 4.9906 |  |
|  |  | Elementary | -.06409 | 2.00113 | 1.000 | -5.7957 | 5.6676 |  |
|  |  | High school | -.51836 | 2.09309 | 1.000 | -6.5134 | 5.4767 |  |
|  |  | Diploma | 1.35718 | 1.68256 | .966 | -3.4620 | 6.1764 |  |
|  |  | Academic | -1.06368 | 1.63092 | .987 | -5.7350 | 3.6076 |  |
|  | High school | Illiteracy | -1.73077 | 2.45436 | .981 | -8.7606 | 5.2990 |  |
|  |  | Elementary | .45427 | 1.90773 | 1.000 | -5.0099 | 5.9184 |  |
|  |  | Middle school | .51836 | 2.09309 | 1.000 | -5.4767 | 6.5134 |  |
|  |  | Diploma | 1.87554 | 1.57032 | .839 | -2.6222 | 6.3733 |  |
|  |  | Academic | -.54532 | 1.51486 | .999 | -4.8842 | 3.7936 |  |
|  | Diploma | Illiteracy | -3.60631 | 2.11515 | .529 | -9.6646 | 2.4519 |  |
|  |  | Elementary | -1.42127 | 1.44548 | .923 | -5.5614 | 2.7189 |  |
|  |  | Middle school | -1.35718 | 1.68256 | .966 | -6.1764 | 3.4620 |  |
|  |  | High school | -1.87554 | 1.57032 | .839 | -6.3733 | 2.6222 |  |
|  |  | Academic | -2.42086 | .86302 | .059 | -4.8927 | .0510 |  |
|  | Academic | Illiteracy | -1.18545 | 2.07431 | .993 | -7.1267 | 4.7558 |  |
|  |  | Elementary | .99959 | 1.38503 | .979 | -2.9674 | 4.9666 |  |
|  |  | Middle school | 1.06368 | 1.63092 | .987 | -3.6076 | 5.7350 |  |
|  |  | High school | .54532 | 1.51486 | .999 | -3.7936 | 4.8842 |  |
|  |  | Diploma | 2.42086 | .86302 | .059 | -.0510 | 4.8927 |  |
| **Job** | Housewife | Employed | -.89194 | 1.06684 | .919 | -3.8159 | 2.0321 |  |
|  |  | Retired | -2.59898 | 1.24870 | .230 | -6.0214 | .8235 |  |
|  |  | Self-employed | -.50461 | 1.04807 | .989 | -3.3772 | 2.3680 |  |
|  |  | Labor | -2.77896 | 1.46065 | .318 | -6.7823 | 1.2244 |  |
|  | Employed | Housewife | .89194 | 1.06684 | .919 | -2.0321 | 3.8159 |  |
|  |  | Retired | -1.70704 | 1.22189 | .630 | -5.0560 | 1.6419 |  |
|  |  | Self-employed | .38733 | 1.01597 | .996 | -2.3973 | 3.1719 |  |
|  |  | Labor | -1.88703 | 1.43779 | .684 | -5.8278 | 2.0537 |  |
|  | Retired | Housewife | 2.59898 | 1.24870 | .230 | -.8235 | 6.0214 |  |
|  |  | Employed | 1.70704 | 1.22189 | .630 | -1.6419 | 5.0560 |  |
|  |  | Self-employed | 2.09437 | 1.20553 | .412 | -1.2098 | 5.3985 |  |
|  |  | Labor | -.17998 | 1.57745 | 1.000 | -4.5035 | 4.1435 |  |
|  | Self-employed | Housewife | .50461 | 1.04807 | .989 | -2.3680 | 3.3772 |  |
|  |  | Employed | -.38733 | 1.01597 | .996 | -3.1719 | 2.3973 |  |
|  |  | Retired | -2.09437 | 1.20553 | .412 | -5.3985 | 1.2098 |  |
|  |  | Labor | -2.27436 | 1.42392 | .500 | -6.1771 | 1.6283 |  |
|  | labor | Housewife | 2.77896 | 1.46065 | .318 | -1.2244 | 6.7823 |  |
|  |  | Employed | 1.88703 | 1.43779 | .684 | -2.0537 | 5.8278 |  |
|  |  | Retired | .17998 | 1.57745 | 1.000 | -4.1435 | 4.5035 |  |
|  |  | Self-employed | 2.27436 | 1.42392 | .500 | -1.6283 | 6.1771 |  |
| **Duration of diabetes** | ≤ 5 | 6-10 | 1.26424 | .90101 | .340 | -.8563 | 3.3848 |  |
|  |  | >10 | .83838 | .91538 | .631 | -1.3160 | 2.9928 |  |
|  | 6-10 | ≤ 5 | -1.26424 | .90101 | .340 | -3.3848 | .8563 |  |
|  |  | >10 | -.42585 | 1.01419 | .907 | -2.8128 | 1.9611 |  |
|  | >10 | ≤ 5 | -.83838 | .91538 | .631 | -2.9928 | 1.3160 |  |
|  |  | 6-10 | .42585 | 1.01419 | .907 | -1.9611 | 2.8128 |  |
| **Method of obtaining health information** | Physician/ Health care providers | Internet | .82297 | 1.02379 | .985 | -2.2113 | 3.8573 |  |
|  |  | Newspapers/magazines | 1.85500 | 1.94313 | .963 | -3.9040 | 7.6140 |  |
|  |  | Friends and acquaintances | 2.10425 | 1.28863 | .661 | -1.7150 | 5.9235 |  |
|  |  | Book | .96575 | 2.04718 | .999 | -5.1017 | 7.0332 |  |
|  |  | Radio, television and satellite | 3.74282^*^ | 1.15041 | .021 | .3332 | 7.1524 |  |
|  |  | I dont Know | 1.05789 | 2.04718 | .999 | -5.0095 | 7.1253 |  |
|  | Internet | Physician/ Health care providers | -.82297 | 1.02379 | .985 | -3.8573 | 2.2113 |  |
|  |  | Newspapers/magazines | 1.03203 | 1.87094 | .998 | -4.5131 | 6.5771 |  |
|  |  | Friends and acquaintances | 1.28128 | 1.17696 | .931 | -2.2070 | 4.7696 |  |
|  |  | Book | .14277 | 1.97879 | 1.000 | -5.7220 | 6.0075 |  |
|  |  | Radio, television and satellite | 2.91985 | 1.02379 | .068 | -.1145 | 5.9541 |  |
|  |  | I dont Know | .23492 | 1.97879 | 1.000 | -5.6298 | 6.0997 |  |
|  | Newspapers/ magazines | Physician/ Health care providers | -1.85500 | 1.94313 | .963 | -7.6140 | 3.9040 |  |
|  |  | Internet | -1.03203 | 1.87094 | .998 | -6.5771 | 4.5131 |  |
|  |  | Friends and acquaintances | .24926 | 2.02801 | 1.000 | -5.7614 | 6.2599 |  |
|  |  | Book | -.88925 | 2.57744 | 1.000 | -8.5283 | 6.7498 |  |
|  |  | Radio, television and satellite | 1.88782 | 1.94313 | .960 | -3.8712 | 7.6469 |  |
|  |  | I dont Know | -.79711 | 2.57744 | 1.000 | -8.4361 | 6.8419 |  |
|  | Friends and acquaintances | Physician/ Health care providers | -2.10425 | 1.28863 | .661 | -5.9235 | 1.7150 |  |
|  |  | Internet | -1.28128 | 1.17696 | .931 | -4.7696 | 2.2070 |  |
|  |  | Newspapers/magazines | -.24926 | 2.02801 | 1.000 | -6.2599 | 5.7614 |  |
|  |  | Book | -1.13851 | 2.12792 | .998 | -7.4452 | 5.1682 |  |
|  |  | Radio, television and satellite | 1.63857 | 1.28863 | .865 | -2.1807 | 5.4578 |  |
|  |  | I dont Know | -1.04636 | 2.12792 | .999 | -7.3531 | 5.2604 |  |
|  | Book | Physician/ Health care providers | -.96575 | 2.04718 | .999 | -7.0332 | 5.1017 |  |
|  |  | Internet | -.14277 | 1.97879 | 1.000 | -6.0075 | 5.7220 |  |
|  |  | Newspapers/magazines | .88925 | 2.57744 | 1.000 | -6.7498 | 8.5283 |  |
|  |  | Friends and acquaintances | 1.13851 | 2.12792 | .998 | -5.1682 | 7.4452 |  |
|  |  | Radio, television and satellite | 2.77708 | 2.04718 | .824 | -3.2904 | 8.8445 |  |
|  |  | I dont Know | .09215 | 2.65677 | 1.000 | -7.7820 | 7.9663 |  |
|  | Radio, television and satellite | Physician/ Health care providers | -3.74282^*^ | 1.15041 | .021 | -7.1524 | -.3332 |  |
|  |  | Internet | -2.91985 | 1.02379 | .068 | -5.9541 | .1145 |  |
|  |  | Newspapers/magazines | -1.88782 | 1.94313 | .960 | -7.6469 | 3.8712 |  |
|  |  | Friends and acquaintances | -1.63857 | 1.28863 | .865 | -5.4578 | 2.1807 |  |
|  |  | Book | -2.77708 | 2.04718 | .824 | -8.8445 | 3.2904 |  |
|  |  | I dont Know | -2.68493 | 2.04718 | .846 | -8.7524 | 3.3825 |  |
|  | I do not know | Physician/ Health care providers | -1.05789 | 2.04718 | .999 | -7.1253 | 5.0095 |  |
|  |  | Internet | -.23492 | 1.97879 | 1.000 | -6.0997 | 5.6298 |  |
|  |  | Newspapers/magazines | .79711 | 2.57744 | 1.000 | -6.8419 | 8.4361 |  |
|  |  | Friends and acquaintances | 1.04636 | 2.12792 | .999 | -5.2604 | 7.3531 |  |
|  |  | Book | -.09215 | 2.65677 | 1.000 | -7.9663 | 7.7820 |  |
|  |  | Radio, television and satellite | 2.68493 | 2.04718 | .846 | -3.3825 | 8.7524 |  |
| **Method of obtaining information related to mental illness** | Physician/ Health care providers | Psychologist/Psychiatrist | 1.65646 | 1.84002 | .946 | -3.6208 | 6.9337 |  |
|  |  | Friends and acquaintances | -1.36072 | 1.46655 | .939 | -5.5669 | 2.8454 |  |
|  |  | Book | -.42700 | 2.52728 | 1.000 | -7.6754 | 6.8214 |  |
|  |  | Internet | .88521 | 1.04029 | .958 | -2.0984 | 3.8688 |  |
|  |  | Radio, television and satellite, TV | 3.72642 | 1.38071 | .078 | -.2335 | 7.6864 |  |
|  | Psychologist/Psychiatrist | Physician/ Health care providers | -1.65646 | 1.84002 | .946 | -6.9337 | 3.6208 |  |
|  |  | Friends and acquaintances | -3.01719 | 2.04151 | .679 | -8.8723 | 2.8380 |  |
|  |  | Book | -2.08346 | 2.89899 | .980 | -10.3979 | 6.2310 |  |
|  |  | Internet | -.77125 | 1.76045 | .998 | -5.8203 | 4.2778 |  |
|  |  | Radio, television and satellite, TV | 2.06996 | 1.98075 | .902 | -3.6109 | 7.7508 |  |
|  | Friends and acquaintances | Physician/ Health care providers | 1.36072 | 1.46655 | .939 | -2.8454 | 5.5669 |  |
|  |  | Psychologist/Psychiatrist | 3.01719 | 2.04151 | .679 | -2.8380 | 8.8723 |  |
|  |  | Book | .93373 | 2.67754 | .999 | -6.7456 | 8.6131 |  |
|  |  | Internet | 2.24594 | 1.36540 | .569 | -1.6701 | 6.1620 |  |
|  |  | Radio, television and satellite, TV | 5.08714^*^ | 1.63965 | .025 | .3845 | 9.7898 |  |
|  | Book | Physician/ Health care providers | .42700 | 2.52728 | 1.000 | -6.8214 | 7.6754 |  |
|  |  | Psychologist/Psychiatrist | 2.08346 | 2.89899 | .980 | -6.2310 | 10.3979 |  |
|  |  | Friends and acquaintances | -.93373 | 2.67754 | .999 | -8.6131 | 6.7456 |  |
|  |  | Internet | 1.31221 | 2.46995 | .995 | -5.7717 | 8.3962 |  |
|  |  | Radio, television and satellite, TV | 4.15342 | 2.63151 | .613 | -3.3939 | 11.7007 |  |
|  | Internet | Physician/ Health care providers | -.88521 | 1.04029 | .958 | -3.8688 | 2.0984 |  |
|  |  | Psychologist/Psychiatrist | .77125 | 1.76045 | .998 | -4.2778 | 5.8203 |  |
|  |  | Friends and acquaintances | -2.24594 | 1.36540 | .569 | -6.1620 | 1.6701 |  |
|  |  | Book | -1.31221 | 2.46995 | .995 | -8.3962 | 5.7717 |  |
|  |  | Radio, television and satellite, TV | 2.84120 | 1.27275 | .226 | -.8091 | 6.4915 |  |
|  | Radio, television and satellite | Physician/ Health care providers | -3.72642 | 1.38071 | .078 | -7.6864 | .2335 |  |
|  |  | Psychologist/Psychiatrist | -2.06996 | 1.98075 | .902 | -7.7508 | 3.6109 |  |
|  |  | Friends and acquaintances | -5.08714^*^ | 1.63965 | .025 | -9.7898 | -.3845 |  |
|  |  | Book | -4.15342 | 2.63151 | .613 | -11.7007 | 3.3939 |  |
|  |  | Internet | -2.84120 | 1.27275 | .226 | -6.4915 | .8091 |  |
| *. The mean difference is significant at the 0.05 level. | | | | | | | | |
